# Engineering Fusion Proteins for Nanomedicine-Based Cytokine Therapy

**DOI:** 10.1021/acs.bioconjchem.5c00182

**Published:** 2025-07-11

**Authors:** Anne de Dreu, Koen de Bruin, Ayla M. Hokke, David P. Schrijver, Danyel N. H. Beelen, Lars M. Verhalle, Maria C. Clavijo Perez, Tom Anbergen, Iris Versteeg, Rianne Maas, Robby C. Zwolsman, Cristina Grao-Roldán, Branca Bartelet, Mirre M. Trines, Daniek Hoorn, Gijs Ros, Yohana C. Toner, Ewelina Kluza, Thijs Beldman, Carlos Pérez-Medina, Mihai G. Netea, Maarten Merkx, Roy van der Meel, Willem J. M. Mulder

**Affiliations:** † Laboratory of Chemical Biology, Department of Biomedical Engineering, 5925 Eindhoven University of Technology, Eindhoven 5600 MB, the Netherlands; ‡ Institute for Complex Molecular Systems (ICMS), Eindhoven University of Technology, Eindhoven 5600 MB, the Netherlands; § Department of Internal Medicine and Radboud Center for Infectious Diseases (RCI), 6034 Radboud University Medical Center, Nijmegen 6525 GA, the Netherlands; ∥ Radboud Institute for Molecular Life Sciences, Radboud University Medical Center, Nijmegen 6525 GA, the Netherlands; ⊥ Centro Nacional de Investigaciones Cardiovasculares (CNIC), Madrid 28029, Spain; # Department of Immunology and Metabolism, Life and Medical Sciences Institute, University of Bonn, Bonn 53115, Germany

## Abstract

Cytokines play a crucial role in cell communication and immunity, making them interesting potential therapeutics for immune-mediated conditions. However, cytokine therapeutics’ clinical translation is hampered by their short blood half-lives and unfavorable biodistribution, resulting in toxicity and poor pharmacokinetics. In this study, we present a strategy to improve cytokines’ pharmacokinetic profile by engineering fusions of apolipoproteins and cytokines, which are formulated into apolipoprotein-based nanoparticles (cytokine-aNPs). After establishing chemical and recombinant fusion approaches, we created a small library of diverse proteins, comprising fusions between apolipoprotein A1 or apolipoprotein E with either interleukin 1β, interleukin 2, or interleukin 4. Although chemical conjugation successfully generated biologically active fusion proteins, their yield and purity were insufficient for cytokine-aNP formulation. Using the recombinant method, we expressed and purified the fusion proteins and then incorporated them into cytokine-aNPs. In addition, we show that all cytokine-aNPs remain stable over at least 10 days and are of similar size and shape. We found that the fusion protein’s cytokine component remains biologically active after purification and after formulation into cytokine-aNPs. In mice, using zirconium-89 radiolabeling to enable *in vivo* positron emission tomography imaging, we found that the pharmacokinetic profile of the cytokines incorporated into aNPs changed considerably. As compared to the native cytokines, we found the cytokine-aNPs to predominantly accumulate in the spleen, bone marrow, lymph nodes, and liver. Together, our results demonstrate that we can improve cytokines’ *in vivo* properties using our fusion protein technology and aNP platform, opening up a translational avenue for nanomedicine-based cytokine therapy.

## Graphical abstract

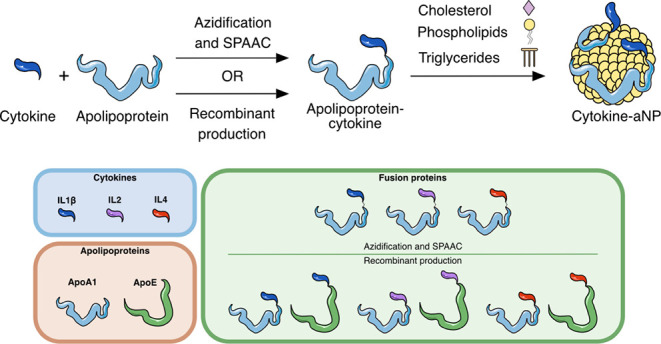

## Introduction

Communication and cooperation between cell subsets are essential for our immune system’s function. The small proteins that facilitate this communication are cytokines. Cytokines are produced and secreted mainly by immune cells and induce signaling through binding to their receptors on a target cell.^1,2^ Cytokine types, receptors, and functions are diverse,^3^ and our immune system relies on the complex interplay between them to facilitate homeostasis, tissue repair, and protection against invading pathogens.^4^ Cytokines are typically distinguished according to their function. Proinflammatory cytokines can activate immunostimulatory programs and activate antipathogenic pathways, typically heightening the inflammatory state.^5^ Anti-inflammatory cytokines lessen or resolve inflammation and often play roles in wound healing.^6,7^

As our immune system plays a crucial role in many different diseases, immunotherapy using cytokines has been extensively explored as a potential treatment strategy.^8–10^ Cytokines currently used in the clinic include interferon α^11^ and interleukin 2 (IL2).^12^ While successfully applied in the treatment of autoimmune diseases and cancer, their use is associated with serious adverse effects, such as hematological toxicity, arrhythmias, and chest pain.^12,13^ These adverse effects are primarily caused by two factors: cytokines’ poor pharmacokinetic profile^14^ and their pleiotropic nature.^9,15,16^

For these reasons, several strategies have been developed to increase cytokine-based drugs’ safety and efficacy profiles. To increase the blood half-life, cytokines have been functionalized with polyethylene glycol (PEG), mutated cytokine variants have been created, and computational modeling is increasingly being applied to design *de novo* proteins. Although the addition of PEG to IL2 significantly increased circulation time, it failed to reduce toxicity.^17,18^ The creation of *de novo* cytokines or the mutation of existing cytokines has resulted in reduced toxicities but has not prolonged these proteins’ blood half-lives.^19^ A promising strategy that tackles both problems is the fusion of cytokines to antibodies, known as immunocytokines. By leveraging the antibody’s long circulation time and target specificity, this approach has led to multiple clinical trials.^20,21^ However, a limitation of these constructs is the potential presence of immunogenic epitopes, which can result in unwanted immune responses.^22^

We previously introduced a strategy to improve the *in vivo* behavior of interleukin 4 (IL4)^23^ by fusing it to apolipoprotein A1 (apoA1) and subsequently incorporating this fusion protein into apolipoprotein-based nanoparticles (aNPs). In mice and nonhuman primates, we found IL4-aNPs to preferentially accumulate in hematopoietic organs and associate with myeloid cells.^23^ To expand on and generalize this concept, we developed both chemical and recombinant engineering strategies for a variety of cytokine-apolipoprotein fusion proteins that readily integrate into aNPs. Chemical conjugation enables the rapid generation of a fusion protein library from commercially available cytokines and apolipoproteins, making it well-suited for small-scale screening purposes and feasibility studies. Candidates identified through this screen can then be recombinantly engineered to improve yield, purity, and scalability for future clinical translation.

In the current study, we create six different fusion proteins composed of either apoA1 or apolipoprotein E (apoE) fused to diverse cytokines (Figure 1). Similar to apoA1, apoE possesses amphipathic properties required for aNP generation.^24^ With the aim to incorporate a diversity of structures and functions, we selected the diverse cytokines, interleukin 1β (IL1β), IL2, and IL4. While IL2 has a similar tertiary structure to IL4, consisting of mostly helical structures,^25,26^ IL1β is composed of mostly beta-strands.^27^ We also sought to use cytokines with diverse functions. IL4 is an anti-inflammatory cytokine,^28^ while IL1β and IL2 have pro-inflammatory properties.^29,30^

**Figure 1:**
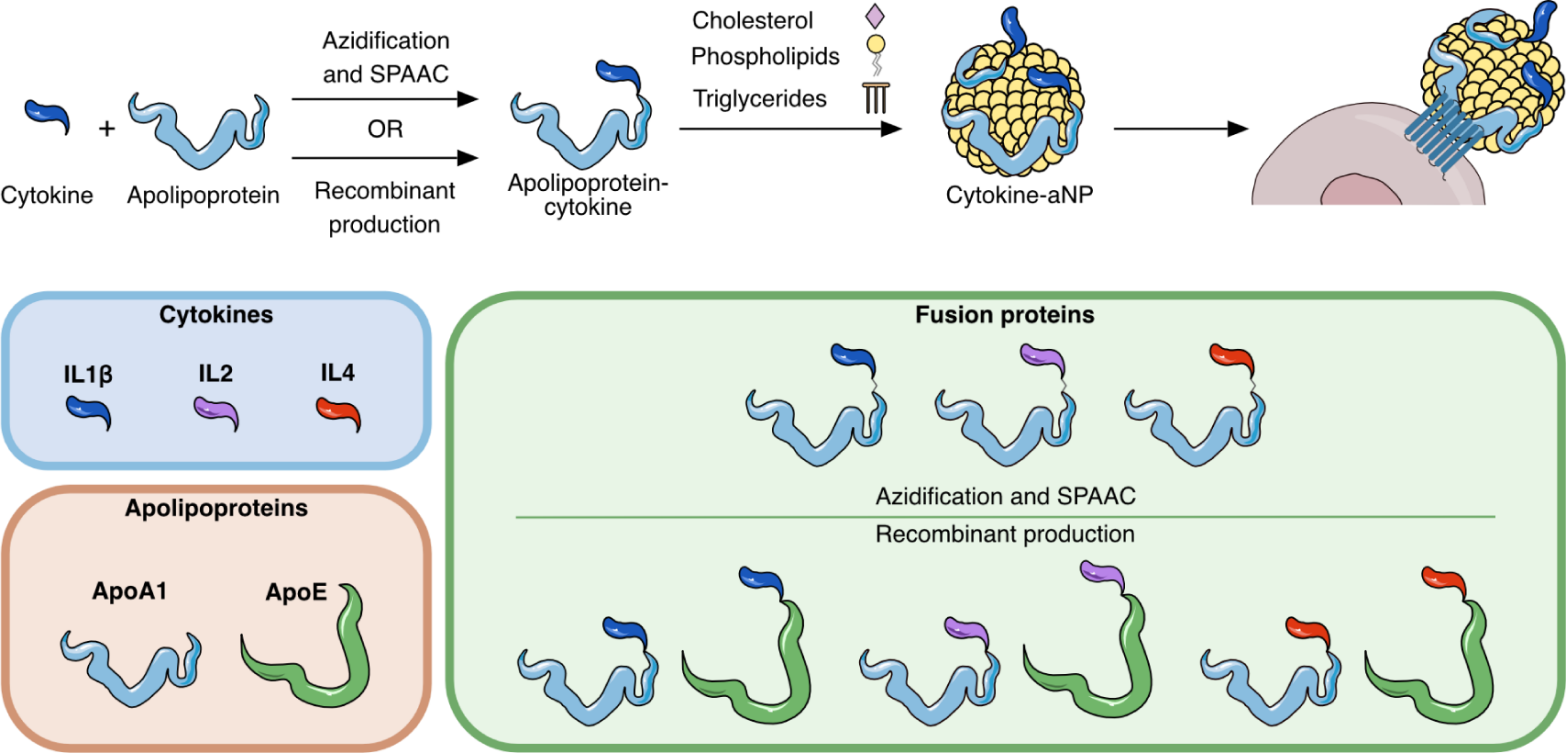
Top panel: Schematic overview of the engineering of apolipoprotein-based cytokine fusion proteins and cytokine-aNPs. Bottom panel: different proteins used and fusion proteins that were created.

Here, we show that we can produce these fusion proteins either through chemical conjugation or recombinant expression in mammalian cells. Both methods generate biologically active fusion proteins, although scalability and purification issues rendered the chemical conjugation strategy unsuitable for aNP formulation. The recombinantly engineered fusion proteins were used to formulate biologically active aNPs. After radiolabeling of the native cytokines, cytokine fusion proteins, and cytokine-aNPs, we performed *in vivo* positron emission tomography in combination with computed tomography (PET-CT) and *ex vivo* gamma counting studies in mice.

## Results and Discussion

### Chemically Conjugated Cytokine-Fusion Protein Production

For the conjugation of IL1β, IL2, or IL4 to apoA1, we applied site-selective, strain-promoted azide–alkyne cycloaddition (SPAAC). To facilitate this reaction, an azide moiety needs to be present on the cytokine. We adapted the aqueous diazotransfer method described by Schoffelen et al., which uses hydrochloric salt of imidazole-1-sulfonyl azide to site-specifically introduce the azide on the N-terminus of a protein.

We applied this Cu­(II)-free, pH 8.5 azidification strategy to the conjugation of cytokines to apoA1. To make the diazotransfer method more widely applicable, we adapted the protocol to be better suited for fragile proteins. Considering that many proteins are not stable when incubated for longer times at room temperature, we investigated performing the reaction at 4 °C (Figure S1). As we did not observe large differences between these conditions, we chose to continue with incubation at 4 °C.

To conjugate the azide-functionalized cytokines to apoA1, we used a mutated version of apoA1 in which a serine in the C-terminal region of the protein was mutated into a cysteine (apoA1-S230C). ApoA1-S230C was recombinantly produced in and purified with immobilized metal affinity chromatography (Figure S2). Subsequently, we functionalized this cysteine-mutated apoA1 with a DBCO-PEG4-maleimide linker and then fused the azidified cytokine to DBCO using regular click-chemistry protocols, using a 1:2 molar ratio of apoA1:cytokine. SDS-PAGE revealed the successful fusion of the different cytokines to apoA1 (Figure 2a). The intensity of the bands on the SDS-PAGE revealed that for all reaction mixtures (lanes 4, 6, and 8 in Figure 2a) the apoA1-cytokine conjugation product made up approximately 30–45%, with apoA1-IL1β making up about 42.9%, apoA1-IL2 32.4%, and apoA1-IL4 35.1% of their respective conjugation mixtures. In each case, about 25% of unconjugated apoA1 remained in the mixture (Table S1). As indicated by the arrows in Figure 2a, there is also a small fraction of apoA1 dimer present in all reaction mixtures, which reacted with the cytokines to form (apoA1)_2_-cytokine conjugation products.

**Figure 2:**
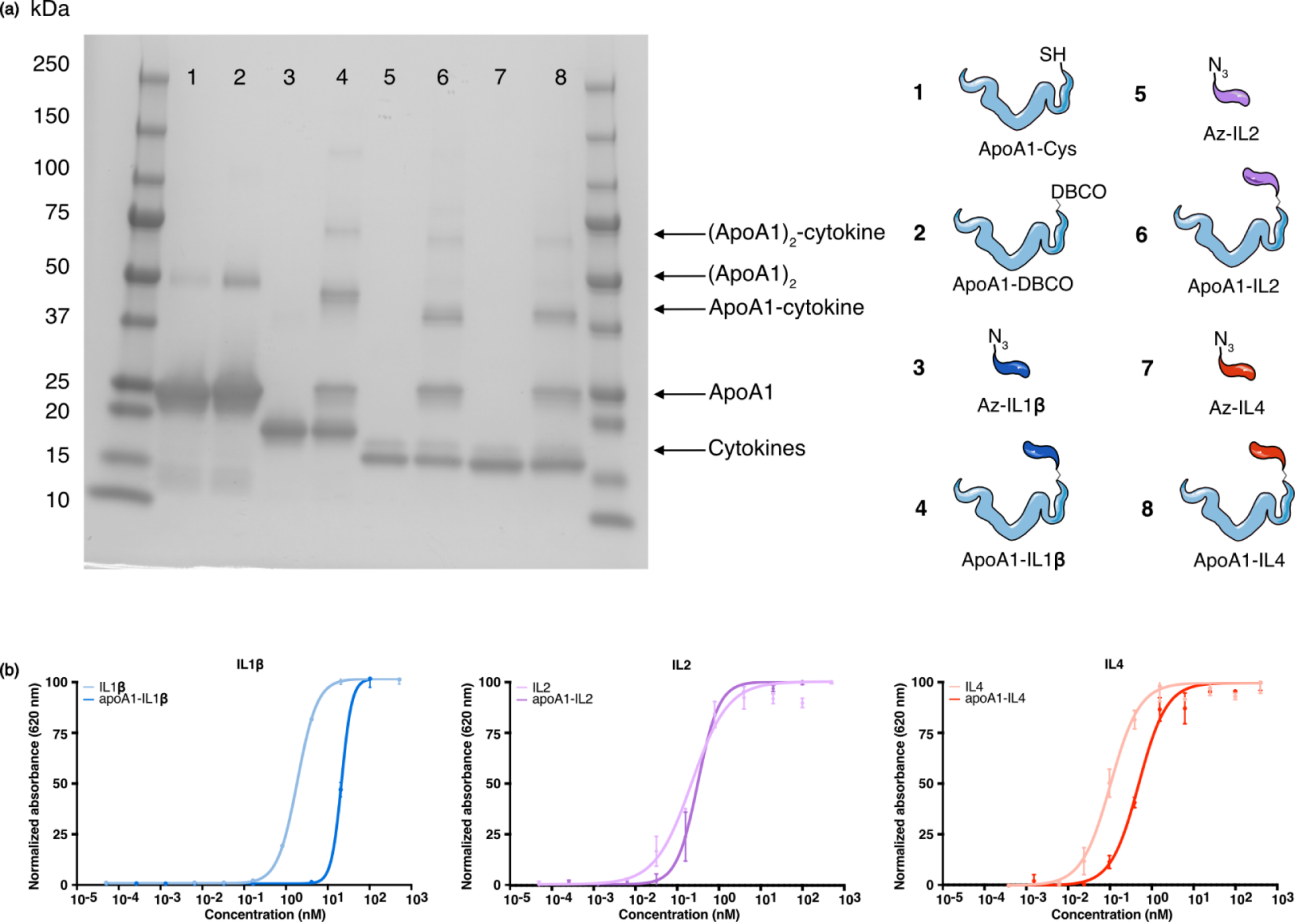
(a) SDS-PAGE of the unpurified reaction mixtures. Numbers indicate the reaction products that were loaded on the gel, and the arrows indicate the different proteins present in these reaction samples. (b) Activation of HEK-Blue reporter cells by the bare cytokine and apoA1-cytokine conjugation products. Data are represented as mean ± s.d.

To remove unconjugated cytokines, we purified the reaction mixture using a Ni-NTA column. The resulting purified mixture showed a reduction in unconjugated cytokine content from 30 to 40% to 15–20% (Table S1 and Figure S3).

Next, we assessed the biological activity of the apoA1-cytokine fusion proteins using a HEK-Blue reporter assay (Figure 2b). Binding of the cytokine to its target receptor, expressed on the HEK-Blue reporter cell, leads to the production of secreted alkaline phosphatase, which causes a change in absorbance upon the conversion of its substrate. We assessed the biological activity of our conjugation products compared to unmodified cytokines and observed almost fully conserved activity for IL2 and IL4, with values of 0.1–0.5 nM (Table S2). EC50 values of these cytokines and apoA1-cytokine conjugation products are in the same range. ApoA1-IL1β displayed a 10-fold reduced bioactivity compared to the unmodified IL1β but remained biologically active with an EC50 value of 20 nM (Table S2).

Although we developed this site-specific conjugation strategy with the goal to create different apoA1-cytokine fusion proteins to form apolipoprotein nanoparticles with lipids^23^ as potential nanomedicine therapeutic candidates, the scale and yields did not suffice. Therefore, we recombinantly engineered the apolipoprotein-cytokine fusion proteins to be expressed in mammalian expression systems in order to continue investigating their therapeutic potential.

### Recombinant Cytokine-Fusion Protein Production

We designed six different fusion protein constructs, all consisting of a cytokine combined with an apolipoprotein. Specifically, we connected the apolipoprotein to the different cytokines through a flexible glycine–glycine-serine (GGS) linker and flanked by an N-terminal chicken RPTPσ signal for secretion (Tables S4 and S5). The proteins were expressed in human embryonic kidney cells (HEK239S) and purified from the culture medium using a C-terminal Twin-Strep-tag. We confirmed the successful expression of all proteins using SDS-PAGE (Figure 3a). All proteins were of sufficient purity and corresponded to the expected molecular weights (Table S5). Due to their amphiphilic nature, apolipoproteins and their derivatives migrate faster through the gel during gel electrophoresis compared to proteins of similar size, causing the bands on SDS-PAGE to appear slightly lower than the expected molecular weight on the protein standard.^23^

**Figure 3:**
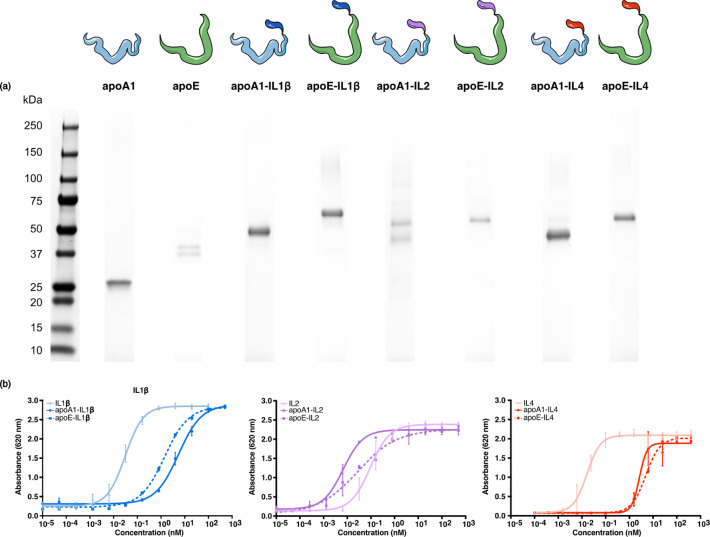
(a) Top: schematic overview of the apolipoproteins and cytokine fusion proteins. Bottom: Coomassie-stained SDS-page gel, showing the presence of bands at the expected molecular weight, indicating successful expression and purification. Expected molecular weights can be found in Table S5. (b) Activation of HEK-Blue reporter cells by the bare cytokine, apoA1-cytokine, and apoE-cytokine fusion protein. Data are represented as mean ± s.d.; individual data points represent triplicate measurements.

We next performed HEK-Blue reporter assays to ensure that the cytokines’ biological activity was preserved after integration into fusion proteins. We observed EC50 values around 0.1 nM for IL2, as well as for the two IL2 fusion proteins. The IL4- and IL1β-based fusion proteins (4.90 and 5.49 nM, respectively) showed an approximate 150× decreased EC50 value compared to the EC50 values of IL4 and IL1β (0.02 and 0.03 nM, respectively) (Table S6). While we observed a decrease in activity for some of the proteins, their EC50 values are in the nanomolar range, indicating that the cytokine is still biologically active (Figure 3b). Taken together, we successfully produced six different biologically active cytokine-fusion proteins.

### Formulation and Characterization of Cytokine-aNPs

To improve the cytokines’ *in vivo* behavior, we formulated the fusion proteins into cytokine-aNPs. This was done by microfluidic mixing of the proteins in an aqueous phase with phospholipids, cholesterol, and triglycerides in an organic phase. The lipids form spherical particles, in which the cytokine-fusion proteins integrate by wrapping around the lipids, providing structural support to the nanoparticles. Dynamic light scattering (DLS) showed that all particles were approximately 60 nm in diameter, with low size dispersity, as indicated by the polydispersity index (PdI) of below 0.2 (Figure 4a). Cryogenic transmission electron microscopy (Cryo-TEM) corroborated this (Figure 4b). We additionally showed that all formulated particles remain stable in PBS for at least 10 days at 4 °C (Figure S4). This highlights the versatility of the aNP platform, as we show that multiple apolipoproteins, as well as differently sized proteins, can be used to form stable nanoparticles.

**Figure 4:**
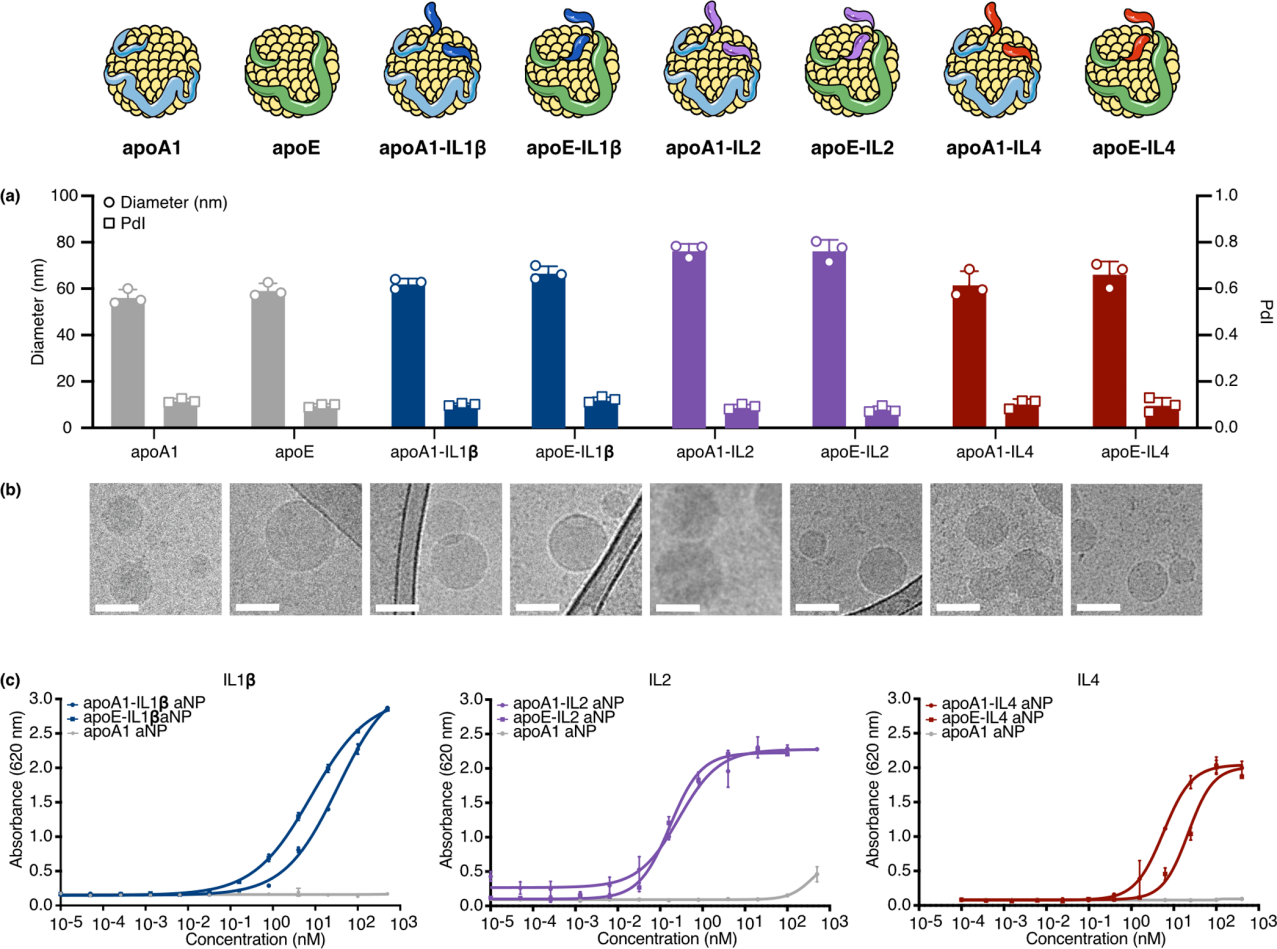
(a) Top: schematic overview of the formulated aNPs. Bottom: DLS results of all formulated aNPs, with on the left *y*-axis the number mean diameter and on the right *y*-axis the PdI. Bars indicate mean ± s.d.; individual data points represent independent triplicates. (b) Cryo-TEM images of all aNPs. Scalebar is 50 nm. (c) Activation of HEK-Blue reporter cells by the cytokine-aNPs and apoA1-aNPs. Data are represented as mean ± s.d.; data points represent triplicate measurements.

We then investigated whether the biological activity of the cytokines was preserved after formulation into aNPs. We again used the HEK-Blue assay for this (Figure 4c). We show that all cytokine-aNPs remain biologically active, albeit with a slightly lower EC50 value compared to the bare cytokine (Table S6). Additionally, nanoparticles containing only apoA1 do not activate the reporter cells, indicating that the observed signal can only be contributed to the cytokine’s activity (Figure 4c).

### *In Vivo* Behavior of Cytokine-aNPs

Next, we set out to study the *in vivo* behavior of the bare cytokines, apolipoprotein-cytokine fusion proteins, and the cytokine-aNPs. We functionalized the proteins with deferoxamine (DFO) and subsequently radiolabeled them by chelating zirconium-89 (^89^Zr) to DFO. We confirmed the successful radiolabeling of the cytokines and cytokine-fusion proteins using radio-SDS-PAGE (Figure S5). We then intravenously administered the radiolabeled cytokines, apolipoprotein-cytokine fusion proteins, and cytokine-aNPs to mice and performed PET-CT imaging at 24 h (Figure 5a,c,e). PET-CT revealed that IL1β and IL2 are cleared rapidly and exclusively through the kidneys, while IL4 appears cleared through both kidneys and liver. The cytokine-apolipoprotein fusion proteins mostly accumulated in the spleen and liver of the mice, as well as the bone marrow. Cytokine-aNPs displayed the strongest accumulation in the bone marrow and lymph nodes, organs rich in immune cells. These results are in line with previous studies^23^ and were also corroborated by *ex vivo* gamma counting of the organs (Figures 5b,d,f and S6). Additionally, similar distribution profiles were obtained for apoA1-aNPs and apoE-aNPs (Figure S7).

**Figure 5:**
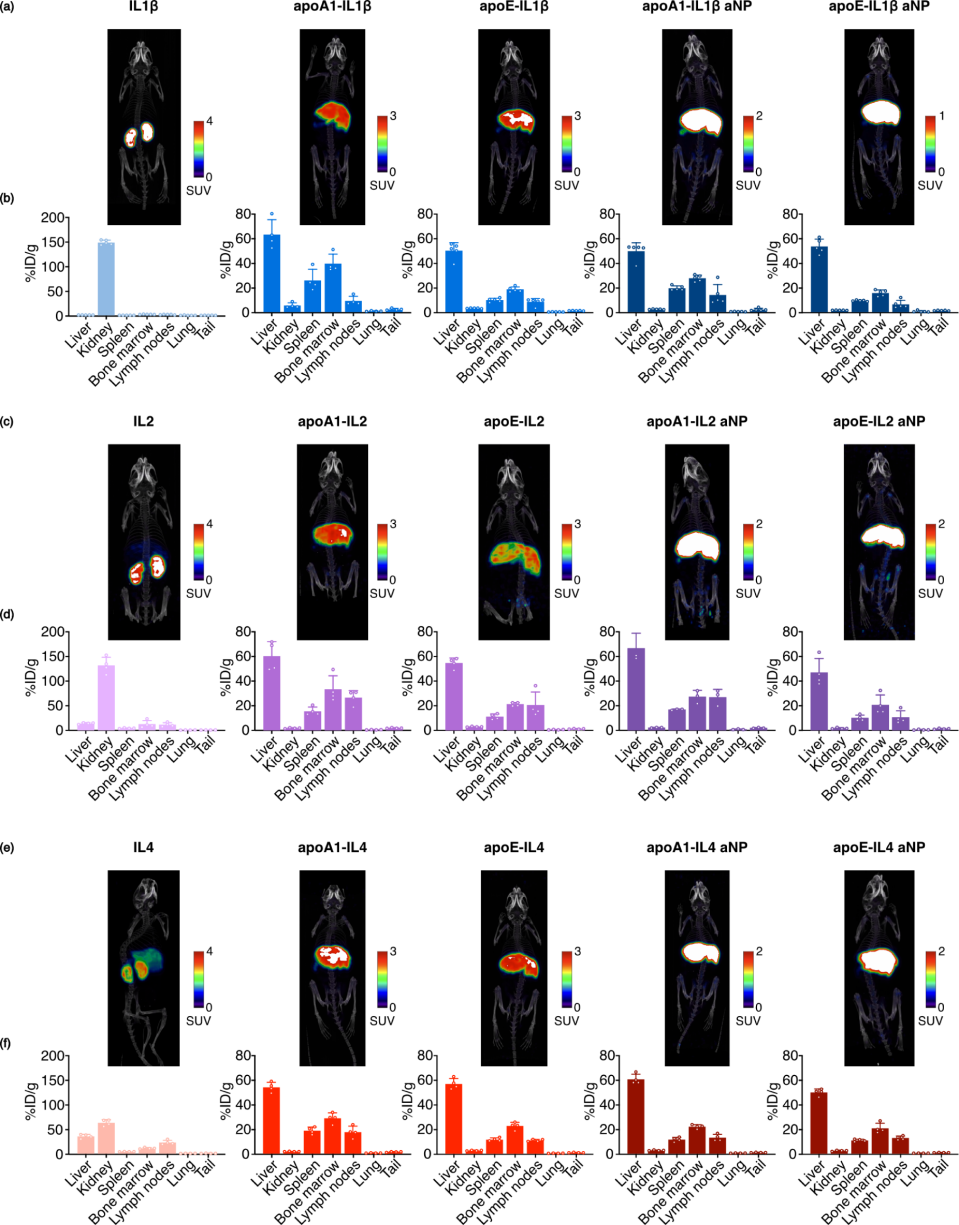
(a,c,e) PET-CT imaging of mice intravenously injected with cytokine, cytokine-apolipoprotein fusion protein, or cytokine-aNP. IL1β, a; IL2, c; IL4, e. (b,d,f) *Ex vivo* gamma counting of pertinent tissues 24 h after injection with proteins or aNPs (*n* = 4) to determine the accumulation of the administered protein or aNP in selected organs. IL1β, (b); IL2, (d), IL4, (f). Bars indicate mean activity ± s.d.; individual data points represent independent replicates.

We then compared the uptake ratios by dividing accumulation in target organs (bone marrow + spleen + lymph nodes) by accumulation in clearance organs (kidney + liver). As anticipated, we found a significant increase in uptake ratio for cytokine-aNP formulations and cytokine fusion proteins compared to bare cytokines (Figure S8). No differences were observed in the apoA1-based constructs versus the apoE-based ones for IL2 and IL4 proteins and formulations. However, for the IL1β fusion proteins and aNPs, we observed a significantly higher uptake ratio for apoA1-based constructs compared to the apoE-based constructs (Figure S8a).

Incentivized by these differences, we investigated the pharmacokinetic profile of the different IL1β constructs. We injected mice with radiolabeled IL1β, IL1β fusion proteins, and IL1β-aNPs and measured the radioactive signal in the blood over time (Figure S9). We observed blood half-lives comparable to those observed for IL4 in previous studies.^23^ Additionally, we found an approximately 2-fold shorter blood half-life for apoE-based constructs compared to apoA1-based constructs (Figure S9b,c). The shorter half-life observed for apoE-based constructs might contribute to the change in uptake ratio of apoE- versus apoA1-based constructs. Taken together, these results indicate the potential of cytokine-apolipoprotein fusion proteins and cytokine-aNPs for improving the pharmacokinetic and biodistribution profiles of the cytokine therapeutics.

### Conclusion

Cytokines are essential for cell communication and immune responses, making them promising potential therapeutics for immune-mediated diseases. Despite this, the clinical application of cytokine-based therapies faces challenges due to their rapid clearance from the bloodstream and poor biodistribution, which leads to suboptimal pharmacodynamics and increased toxicity. To be able to take advantage of cytokine’s therapeutic features, we developed a nanoparticle protein-engineering strategy, thereby overcoming the unfavorable *in vivo* properties of cytokines. We created six different fusion proteins using chemical conjugation or recombinant expression in mammalian cells. We optimized the site-specific aqueous diazotransfer using imidazole-1-sulfonyl azide to be more straightforward and better suited for fragile proteins. We then showed that we can use this technique to integrate azides in cytokines site-specifically at the N-terminus. We next conjugated these azidified cytokines to apoA1-DBCO to create apoA1-cytokine conjugations. This method did not impact the biological activity of the cytokines, again indicating its applicability for fragile proteins. Although we developed this site-specific conjugation strategy in order to create different apoA1-cytokine fusion proteins to form apolipoprotein nanoparticles with lipids^23^ as potential nanomedicine therapeutic candidates, the scale and yields were not sufficient. Therefore, we changed course and expressed six different apolipoprotein-cytokine fusion constructs in mammalian cells. We formulated these proteins into stable aNPs of similar size and shape and showed that the cytokine is biologically active before and after formulation. We additionally show that the *in vivo* distribution profile is improved when cytokines are incorporated into fusion proteins or aNPs. With this study, we show that this platform can be applied to a variety of cytokines and apolipoproteins. This indicates the potential of cytokine-apolipoproteins in the field of cytokine therapeutics.

Compared to the chemical conjugation strategy, the recombinant production of the fusion proteins is much more time-consuming and labor intensive. The chemical modification and conjugation of cytokines or other proteins that are commercially available, and thus do not require in-house expression, would be better suited for the production of libraries of fusion proteins. However, due to the issues related to chemistry, manufacturing, and controls (CMC) and scalability, this is less suitable for further development into a clinically translatable product. Additionally, with the recombinant production in mammalian cells, we anticipate the immunogenicity of the proteins to be lower. This, together with the use of apolipoproteins native to the human body, reduces the chances of developing an immune response against this therapy. Nevertheless, it is well-known that immunogenicity is a challenge for cytokine therapeutics and other biologicals. For example, treatment with the hu14.18-IL2 immunocytokine, a fusion of a humanized antibody and IL2, raises anti-idiotypic antibodies in two-thirds of the patient population.^31^ Therefore, it remains important to study the immunogenicity of these therapeutic proteins.

While we have performed some initial studies on the *in vivo* behavior of the apoA1 and apoE fusion proteins and aNPs, we did not see any major differences between the constructs. For future studies, it would be interesting to investigate the *in vivo* behavior not only at an organ level but also at a cellular level. It is expected that the interaction with myeloid cells is more pronounced for apoA1-based constructs compared to apoE, as the native function of apoA1 is to interact with these cells. For that reason, apoA1-based constructs might be more suitable for applications where the innate immune system needs to be engaged, whereas apoE-based constructs might be more generally applicable. Additionally, apoE-based constructs might be applied for cancer therapy, as the LDL receptor is often overexpressed on tumor cells.^32,33^

We have shown two methods for engineering these fusion proteins. Through azidification and SPAAC, we can quickly create fusion proteins from commercially available sources, making this ideal for library generation or feasibility studies. Due to the issues in purification and scalability, we then diverted to recombinant production of the fusion proteins, which allowed us to scale the production and integrate the proteins in aNPs. We have seen that a wide variety of cytokines can be introduced in this platform. Therefore, the cytokine-nanoparticle technology may find applications in many immune-mediated diseases, ranging from cancer and autoimmunity to sepsis and other inflammatory conditions.

## Experimental Procedures

### Materials

All reagents and solvents were obtained from commercial sources and were used without further purification. All proteins were produced in-house. All DNA and protein sequences can be found in Tables S3–S5.

### Bacterial Expression and Purification of apoA1-S230C, apoA1, and apoE

ClearColi BL21 (DE3) (Lucigen) were transformed with pET20b­(+) expression vectors and inoculated, and protein expression was started according to protocols described in ref 23. Cells were collected by centrifugation at 10,880 *g* and 4 °C for 10 min before preparation of lysates and purification. Cells were lysed using BugBuster Protein Extraction Reagent (Merck) supplemented with Benzonase nuclease (Merck) according to manufacturer’s instructions and incubated on a shaker for 30 min at room temperature. Cell lysates were centrifuged at 4 °C, 39,000 *g*, for 30 min. The filtered supernatant was loaded on an IMAC nickel column and washed with 10 column volumes of wash buffer (0.01 M imidazole, 20 mM Tris, and 0.5 M NaCl at pH 7.9). Proteins were eluted from the column with 0.5 M imidazole, 20 mM Tris, and 0.5 M NaCl at pH 7.9. Eluate was collected, concentrated, and buffer-exchanged to PBS. Purity was assessed using SDS–PAGE, and the protein was snap-frozen in liquid nitrogen prior to storing at −80 °C. Protein mass was confirmed by Q-ToF LC-MS (WatersMassLynx v4.1), using MagTran V1.03 for MS.

### Bacterial Expression and Purification of Cytokines

Shuffle T7 chemically competent were transformed with a pET28a-His_6‑_SUMO-IL4-strep, pET28a-His_6‑_SUMO-IL2-strep, or pET28a-His_6‑_SUMO-IL1β expression vector. Transformed bacteria were inoculated in 40 mL lysogeny broth (Sigma-Aldrich) supplemented with 50 μg/mL kanamycin and grown overnight at 250 rpm and 37 °C. Subsequently, the overnight culture was inoculated in 2YT medium (16 g L-1 peptone, 10 g L-1 yeast extract, and 10 g L-1 NaCl) supplemented with 50 μg/mL kanamycin and incubated at 150 r.p.m. and 37 °C. At an optical density at 600 nm of 0.6, 0.1 mM isopropyl β-d-thiogalacopyranoside was added to induce protein expression. The culture was further incubated at 20 °C at 150 rpm overnight. Bacterial pellets were then obtained by centrifugation at 10,000*g* for 10 min at room temperature. The resulting supernatant was discarded. The pellet obtained from the expression was resuspended in 10 mL extraction buffer (100 mM TRIS, 250 mM NaCl, pH 7.0) per gram of cell pellet. Twenty-five U Benzonase Nuclease (Merck) were then added. One cOmplete Protease Inhibitor Cocktail Tablet was added per 50 mL of extraction buffer. The resulting solution was stirred at 4 °C for 30 min until no clumps remained. The solution was then homogenized three times using the Avestin Emulsiflex C3 at 15,000–20,000 psi while being kept on ice. The cell lysate was then centrifuged at 16,000 *g* for 20 min at 4 °C. The filtered supernatant was purified on an IMAC nickel column as described above. Eluates were collected.

SUMO hydrolase was added to the SUMO-IL-4, SUMO-IL2, and SUMO-IL1β in a ratio of 1 mg hydrolase/500 mg protein. The solution was then dialyzed to PBS (pH 7.0) using a Snakeskin 10 kDa cutoff dialysis bag (Thermo Scientific) while gently stirring at 4 °C overnight. Then the resulting protein solution was centrifuged at 4000 *g* for 20 min, and the supernatant was filtered using a 0.2 μM syringe filter to remove any aggregated protein. The IMAC purification protocol was repeated, and the fractions were again analyzed using SDS-PAGE. Fractions containing IL4, IL2, or IL1β were buffer exchanged to PBS (pH 7.9), and the final concentration was determined using a NanoDrop 1000 spectrophotometer. The proteins were then snap-frozen in liquid nitrogen and stored at −80 °C.

### Azide Introduction into Cytokines

Imidazole-1-sulfonyl azide hydrochloride (Fluorochem) was dissolved in Milli-Q to a concentration of 95 mM. The pH of the azidotransfer solution and the cytokines (PBS) was set to 8.0. Next, the solution was added to the cytokine in a 1:250 cytokine:diazotransfer reagent molar ratio. The reaction mixture was incubated overnight at 4 °C. The following day, the azido-cytokines were desalted using a PD MidiTrap G-25 desalting column (Cytiva).

### Preparation of apoA1-DBCO

DBCO-PEG4-Maleimide (Sigma-Aldrich) was dissolved in DMSO (100 mg/mL). Next, the linker was added to apoA1 S230C (pH 7.5, PBS) in a 1:10 apoA1:linker molar ratio. The reaction mixture was incubated overnight at 4 °C. The following day, the apoA1-DBCO was desalted using a PD MidiTrap G-25 desalting column (Cytiva).

### Conjugation of apoA1 to Cytokines

ApoA1-DBCO was added to the azido-cytokine solution in a 1:2 apoA1:cytokine molar ratio. The reaction mixture was incubated overnight at 4 °C. Purity and successful conjugation were confirmed by SDS-PAGE and Q-ToF LC-MS (WatersMassLynx v4.1), using MagTran V1.03 for MS. The protein was snap-frozen in liquid nitrogen prior to storage at −80 °C.

### Purification of apoA1-Cytokines

After conjugation, reaction mixtures were run through an IMAC column containing immobilized nickel ions. The column was washed with 8 column volumes of buffer A50 (20 mM Tris, 500 mM NaCl, 50 mM imidazole, pH 7.9). To elute the fusion proteins, 8 column volumes of buffer A500 (20 mM Tris, 500 mM NaCl, 500 mM imidazole, pH 7.9) were applied to the column. Flow-through, wash, and eluate were collected and analyzed with SDS-PAGE. Fusion proteins were buffer exchanged to PBS using Amicon Ultracentrifugal Filters (Amicon). They were snap-frozen in liquid nitrogen and stored at −80 °C.

### (Radio-)­SDS-PAGE

To confirm fusion, expression, or radiolabeling of fusion proteins or particles, samples were mixed with SDS loading buffer (100 mM Tris-Cl, 4% sodium dodecyl sulfate, 0.2% bromophenol blue, 20% glycerol, 200 mM DTT, pH 6.8) in 1:1 ratio and heated for 5 min at 95 °C. Next, samples and precision plus protein dual color ladder (Bio-Rad) were loaded in a Mini-PROTEAN TGTTM Precast Gel (Bio-Rad) and ran in Tris/Glycine/SDS running buffer (Bio-Rad). After gel electrophoresis, gels were washed with Milli-Q for 15 min and stained with Bio-Safe Coomassie G-250 Stain (Bio-Rad) for 30 min. The gel was destained with Milli-Q until the bands were visualized using an ImageQuant gel imager (GE Healthcare). To confirm radiolabeling of fusion proteins, between gel electrophoresis and Bio-Safe Coomassie G-250 Stain, the gels were transferred to autoradiography plates and visualized using an Amersham Typhoon Biomolecular Imager (GE Healthcare). The intensity of the bands was determined using ImageJ analysis.

### Mammalian Expression and Purification Fusion Proteins

To produce and purify the lentivirus, HEK293T cells were co-transfected with the second-generation pHR plasmid carrying the desired transgene and the vectors encoding the packaging proteins (pCMVR8.74 and VSV-G envelope pMD2.G) following methods described in ref 23. The resulting virus was used to transduce HEK293S cells in transfection medium (DMEM, 10% HI FBS, 1× Polybrene, Sigma-Aldrich) for 24 h. Subsequently, cells were cultured in expression medium (50% EX-CELL 293 Serum-Free Medium for HEK293 Cells, Merck) and 50% FreeStyle 293 Expression Medium (Thermo Fisher Scientific), supplemented with Glutamax, 1% Pen-Strep and 1 μg ml–1 doxycycline (Merck) on a shaker at 150 r.p.m. for 3 days at 37 °C. Cytokine-fusion proteins were obtained from the culture supernatant following methods described in ref 23. Fusion proteins were concentrated and snap-frozen in liquid nitrogen before being stored at −80 °C.

### Human Embryonic Kidney 293 Reporter Cell Assays

HEK-Blue IL-1β, HEK-Blue IL2, and HEK-Blue IL4/IL13 reporter cells were purchased from InvivoGen. These cell lines have a fully active STAT6 pathway and carry a STAT6-inducible SEAP reporter gene. The HEK-Blue cells produce SEAP in response to the corresponding cytokine. The levels of secreted SEAP can be determined with QUANTI-Blue (InvivoGen). Assays were performed following methods described in ref 23. The obtained data were analyzed using GraphPad Prism 10 software. A dose–response curve fit was performed from which EC50 values were extracted.

### Formulation of aNPs

All phospholipids were purchased from Avanti Polar Lipids. To formulate aNPs, 1-palmitoyl-2-hydroxy-*sn*-glycero-3-phosphocholine (Lyso-PC, 0.18 mg) was dissolved in methanol, 1-palmitoyl-2-oleoyl-*sn*-glycero-3-phosphocholine (POPC, 0.67 mg), tricaprylin (Sigma-Aldrich, 2.66 mg), and cholesterol (Sigma-Aldrich, 0.05 mg) all dissolved in chloroform, were combined in a glass vial, and dried under vacuum. The resulting lipid film was redissolved in ethanol (0.9 mL total volume). Separately, a solution of apolipoprotein or apolipoprotein-cytokine fusion protein in PBS (6.5 mL, 3.67 μM) was prepared. The dissolved lipid film and the diluted protein were simultaneously injected using a microfluidic pump fusion 100 (Chemyx) into a Zeonor herringbone mixer (Microfluidic ChipShop, product code 10000076) with a flow rate of 0.8 mL/min for the lipid solution and a flow rate of 6 mL/min for the protein solution. The obtained solution was filtered through a 0.22 μm PES syringe filter and concentrated by centrifugal filtration using a 100 kDa molecular weight cutoff (MWCO) Vivaspin tube (Sartorius Biotech) at 4000 r.p.m. and 4 °C until a volume of approximately 1 mL remained. PBS (10 mL) was added, and the solution was concentrated to 1 mL; this was repeated twice. The obtained aNP solutions were filtered through a 0.22 μm PES syringe filter and stored at 4 °C. The obtained aNP formulations were analyzed by DLS on a Zetasizer Nano ZSP (Malvern Instruments). Values are reported as the mean number-average size distribution.

### Cryo-TEM

Cryo-TEM imaging was done according to methods described in ref 23.

### Animal Models

Female WT C57BL/6J mice and OT1 C57BL/6-Tg­(TcraTcrb)­1100Mjb/Crl mice (approximately 8–11 weeks old and approximately 20 g) were purchased from Charles River. All animals were cohoused in climate-controlled conditions at 20–24 °C, 45–65% humidity, with 12 h light–dark cycles and provided water ad libitum and fed a standard chow diet. Animal care and experimental procedures were based on approved institutional protocols from Radboud UMC and were conducted in compliance with European and Dutch guidelines according to the care and use of laboratory mice after approval by the Radboud University Medical Center’s Dierexperimentencommissie (DEC) (CCD License: AVD10300 2021 14977).

### Radiolabeling aNPs

For radio labeling, proteins and aNPs were incubated with two molar excesses of DFO-p-NCS (5 mg mL^–1^ in DMSO) at room temperature for 2 h in PBS (pH 8.8) and separated from unreacted DFO-p-NCS via a PD10 desalting column (GE Healthcare). For radiolabeling, DFO-bearing proteins and aNPs were incubated with ^89^Zr at 37 °C using a thermomixer at 600 r.p.m. for 30 min. Radiolabeled aNPs were separated from unbound ^89^Zr via Zeba spin desalting column (Themo Scientific) filtration. Radioactivity was measured after desalting to determine the radiolabeling efficiency.

### Pharmacokinetics and Biodistribution in Mice

C57BL/6 mice were intravenously injected with ^89^Zr-labeled cytokines, cytokine fusion proteins, or cytokine-aNPs. At predetermined time points, 1, 5, 15, and 30 min and 1, 2, 4, and 24 h after injection, radioactivity in the blood was determined according to methods described in ref 23.

### PET-CT Imaging

C57BL/6 mice were intravenously injected with ^89^Zr-labeled cytokines, cytokine fusion proteins, or cytokine-aNPs. After 24 h, mice were anaesthetized using a gas mixture of 2% isoflurane and 5% oxygen. PET-CT imaging was done according to methods described in ref 34. All PET and CT data were processed using OsiriX Medical Imaging software (version 13.0.3)

### Safety Statement

This study involved hazardous chemicals (e.g., DMSO, imidazole-1-sulfonyl azide hydrochloride, and ethanol), which were handled using standard PPE. Biological materials were handled under Biosafety Level 1 (BSL-1) conditions. Lentiviral work was executed under Biosafety Level 2 (BSL-2) conditions. Radiochemistry was performed with the appropriate safety precautions such as lead shields. No unexpected or unusually high hazards were encountered.

## Supplementary Material



## Notes

SDS-PAGE of conjugation products and expressed proteins, DLS measurements of nanoparticles over time; analysis of radiolabeling using radio SDS-PAGE, ex vivo gamma counting of all organs of mice IV injected with cytokines, cytokine fusion proteins and cytokine-aNPs, uptake ratios for cytokines, cytokine fusion proteins, and cytokine-aNPs, blood-half-life results of IL1β, IL1β fusion proteins and IL1β-aNPs, supporting tables with purities of the conjugation mixutures, EC50 values, and all DNA and protein sequences (PDF)

## Abbreviations

IL2: interleukin 2
PEG: polyethylene glycol
IL4: interleukin 4
apoA1: apolipoprotein A1
aNPs: apolipoprotein-based nanoparticles
apoE: apolipoprotein E
IL1β: interleukin 1β
LDL: low-density lipoprotein
GGS: glycine–glycine-serine
HEK2193S: human embryonic kidney cells
SDS-PAGE: sodium dodecyl sulfate-polyacrylamide gel electrophoresis
DLS: dynamic light scattering
^89^Zr: zirconium-89
PET-CT: positron emission tomography with computed tomography
ID%/g: injected dose per gram tissue.
