# Supplementary material for: Engineering Fusion Proteins for Nanomedicine-Based Cytokine Therapy
Source: Bioconjug Chem. 2025 Jul 11;36(8):1698–708. doi: 10.1021/acs.bioconjchem.5c00182 (PMC12371689; doi:10.1021/acs.bioconjchem.5c00182)
Supplement: Supplementary file 1 [file bc5c00182_si_001.pdf]

Supporting information

Engineering Fusion Proteins for Nanomedicine-based Cytokine Therapy

AUTHORS

Anne de Dreu [‡,1,2], Koen de Bruin[‡,1,2], Ayla M. Hokke [‡,1,2], David P. Schrijver[1,2], Danyel N. H. Beelen[1], Lars M. Verhalle[1], Maria C. Clavijo Perez[1], Tom Anbergen[3,4], Iris Versteeg[3,4], Rianne Maas[3,4], Robby C. Zwolsman[1,2], Cristina Grao-Roldán[3,4,5], Branca Bartelet[3,4], Mirre M. Trines[1,2], Daniek Hoorn[1,2], Gijs Ros[3,4], Yohana C. Toner[3,4], Ewelina Kluza[1,2], Thijs Beldman[3,4], Carlos Pérez-Medina[5], Mihai G. Netea[3,6], Maarten Merkx[1,2], Roy van der Meel[1,2], Willem J. M. Mulder[1-4*]

AUTHOR AFFILIATIONS

[1]Laboratory of Chemical Biology, Department of Biomedical Engineering, Eindhoven University of Technology, Eindhoven, the Netherlands.

[2]Institute for Complex Molecular Systems (ICMS), Eindhoven University of Technology, Eindhoven, the Netherlands.

[3]Department of Internal Medicine and Radboud Center for Infectious Diseases (RCI), Radboud University Medical Center, Nijmegen, the Netherlands.

[4]Radboud Institute for Molecular Life Sciences, Radboud University Medical Center, Nijmegen, the Netherlands

[5]Centro Nacional de Investigaciones Cardiovasculares (CNIC), Madrid, Spain.

[6]Department of Immunology and Metabolism, Life and Medical Sciences Institute, University of Bonn, Bonn, Germany

[‡]These authors contributed equally

[*]Corresponding author

SUPPLEMENTARY FIGURES

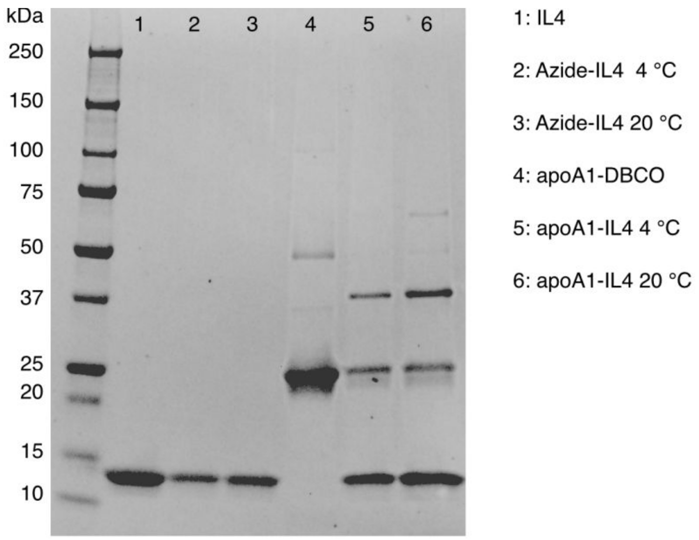

Supplementary Figure 1. SDS-PAGE comparing the introduction of an azide in IL4 at 4 °C or at 20 °C, and subsequent conjugation to apoA1-DBCO.

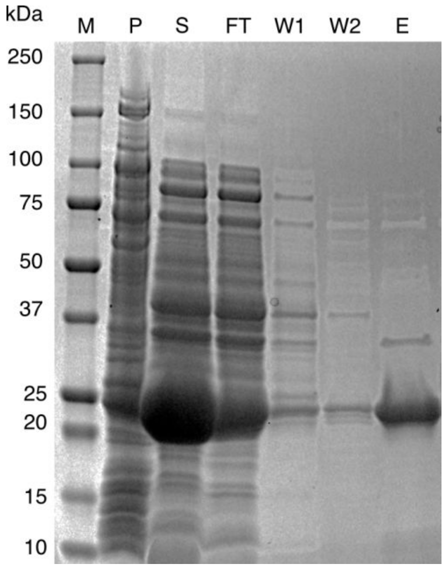

Supplementary Figure 2. SDS-PAGE analysis of cysteine-modified apoA1 (apoA1-S230C). P: pellet, SN: supernatant, FT: flow-through, W1: wash 1, W2: wash 2, E: elution fraction, M: marker.

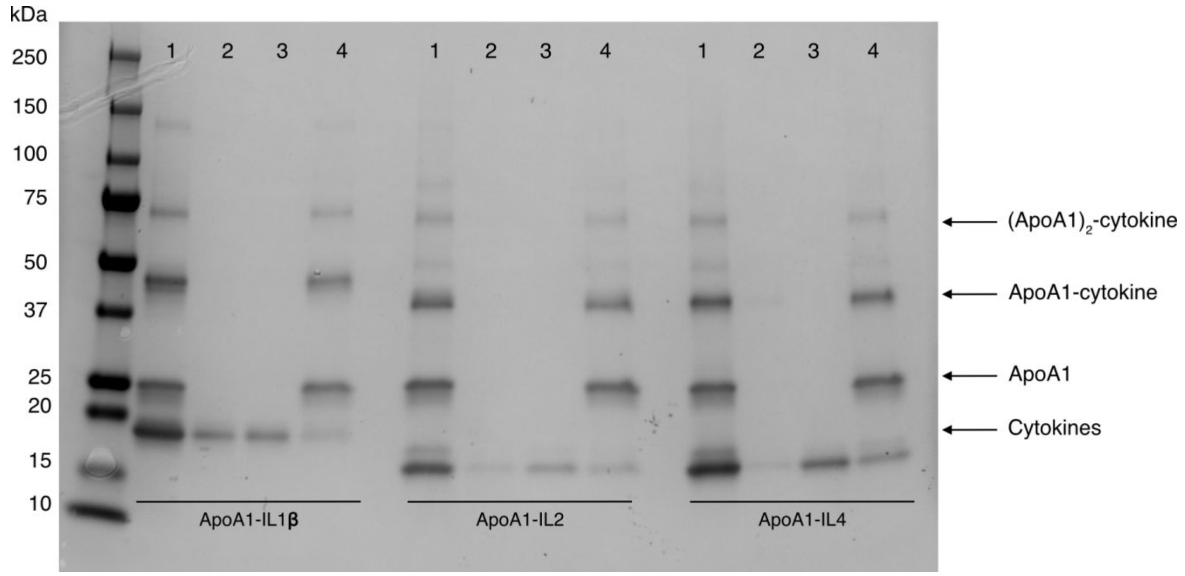

Supplementary Figure 3. SDS-PAGE of the Ni-NTA purification of different fusion-proteins. Sample type is indicated at the bottom of the gel, arrows indicate the different proteins present in the samples. 1: reaction product, 2: flow-through, 3: wash, 4: elution.

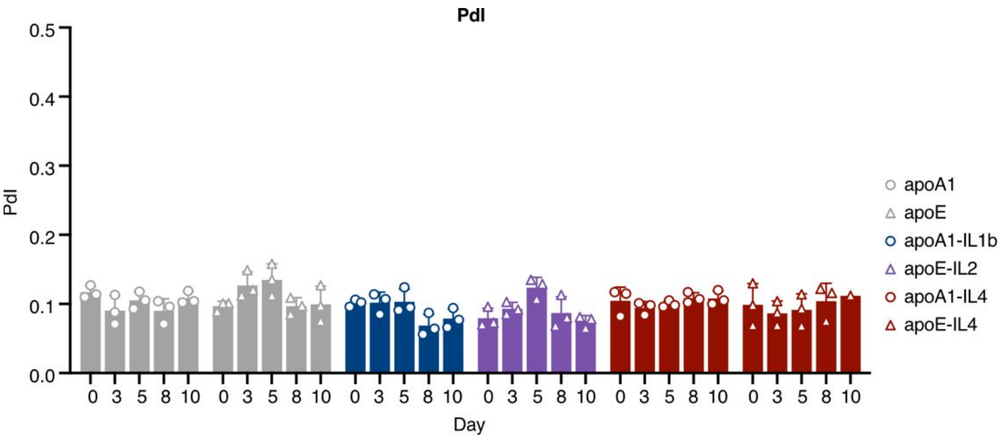

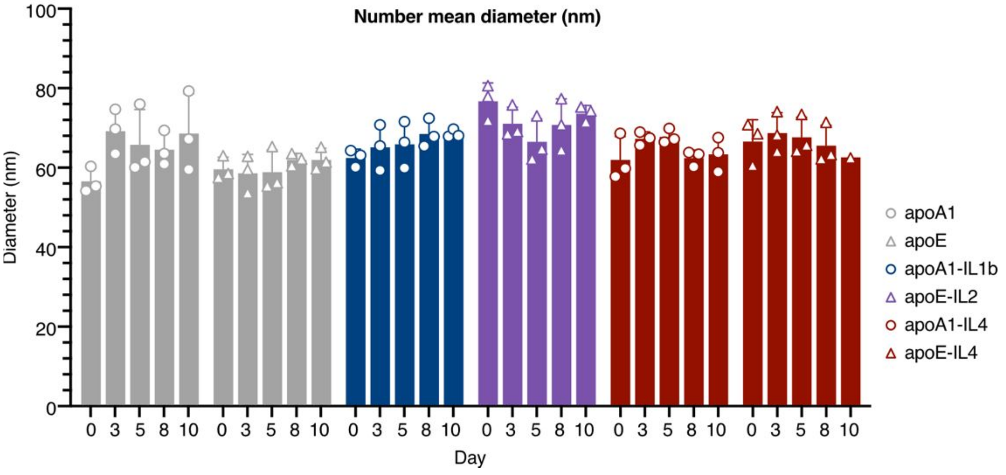

Supplementary Figure 4. DLS measurements over time for diverse cytokine-aNPs. Top: PdI. Bottom: number mean diameter.

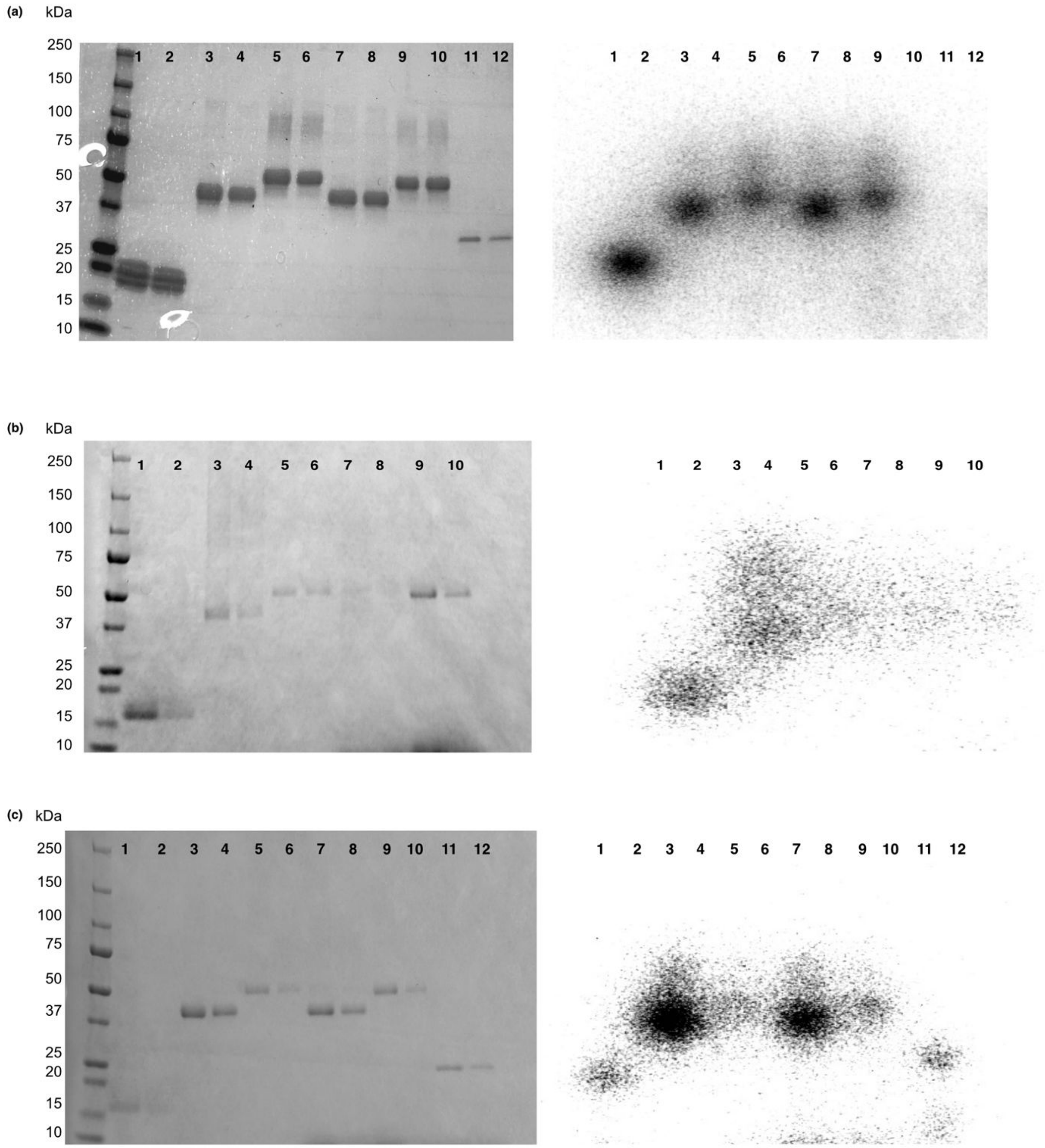

Supplementary Figure 5. SDS-PAGE (left) and autoradiography of SDS-PAGE (right) of cytokines, cytokine fusion proteins and cytokine aNPs. Uneven lanes are radiolabeled, even lanes are not. (a) IL1β. 1+2, IL1β; 3+4 apoA1-IL1β; 5+6, apoE-IL1β; 7+8, apoA1-IL1β aNP; 9+10, apoE-IL1β aNP; 11+12, apoE aNP (b) IL2. 1+2, IL2; 3+4 apoA1-IL2; 5+6, apoE-IL2; 7+8, apoA1-IL2 aNP; 9+10, apoE-IL2 aNP. (c) IL4. 1+2, IL4; 3+4 apoA1-IL4; 5+6, apoE-IL4; 7+8, apoA1-IL4 aNP; 9+10, apoE-IL4 aNP; 11+12, apoA1 aNP.

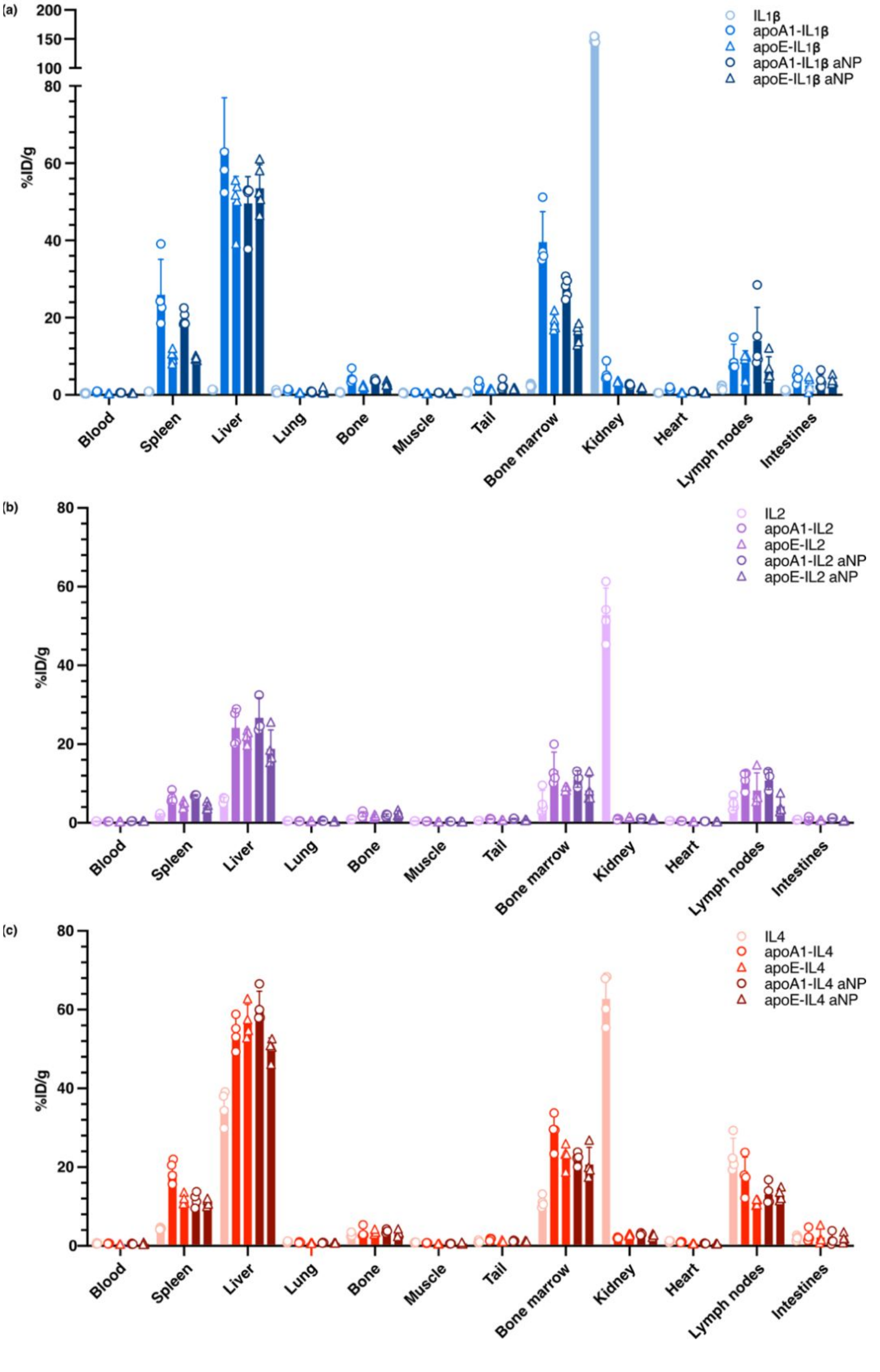

Supplementary Figure 6. *Ex vivo* gamma counting of organs from mice intravenously injected with cytokines, cytokine fusion proteins and cytokine-aNPs. (a) I1b constructs. (b) IL2 constructs. (c) IL4 constructs.

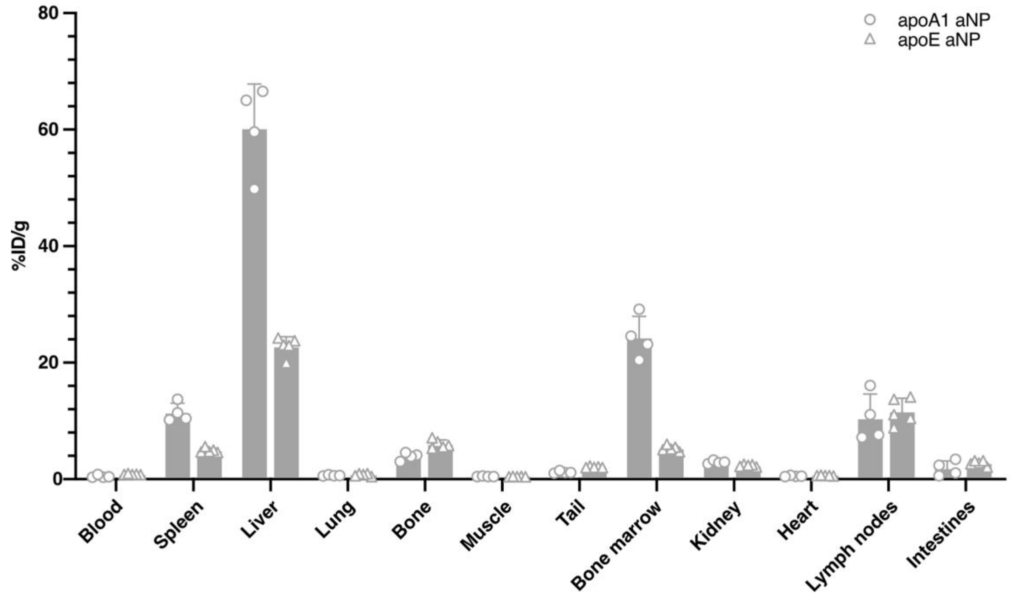

Supplementary Figure 7. *Ex vivo* gamma counting of organs from mice intravenously injected with apoA1-aNPs or apoE-aNPs.

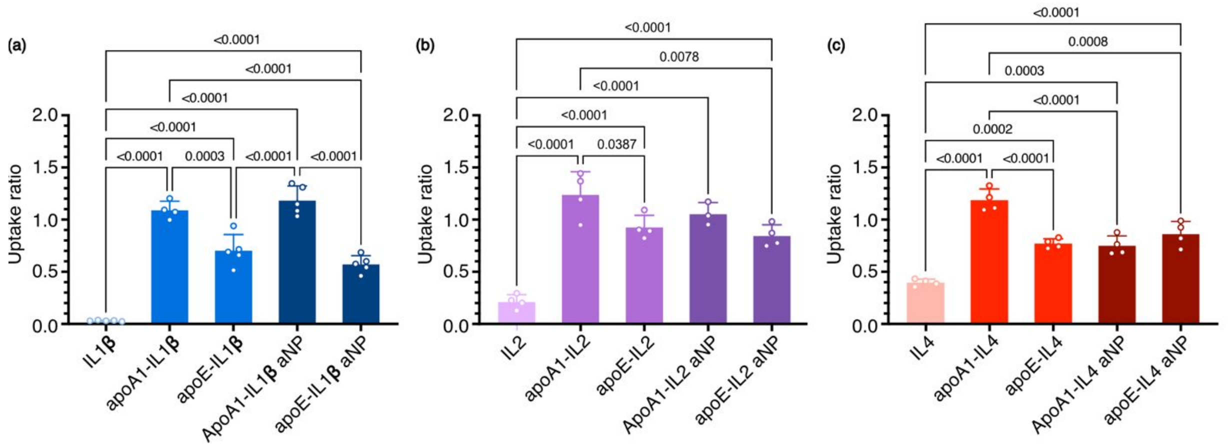

Supplementary Figure 8. Uptake ratio calculated by dividing the uptake in target organs (spleen, bone marrow and lymph nodes) by the clearance organs (liver, kidneys). Calculated based on ID%/g, 24 hours after intravenous injection of bare cytokines, cytokine fusion proteins and cytokine-aNPs. Data are presented as mean ± SD. Significance (one-way ANOVA (F value = 12.21) with Tukey's post-hoc analysis) is noted above bars. (a) IL1β constructs. F value = 89.10. (b) IL2 constructs F value = 32.48. (c) IL4 constructs. F value = 42.16.

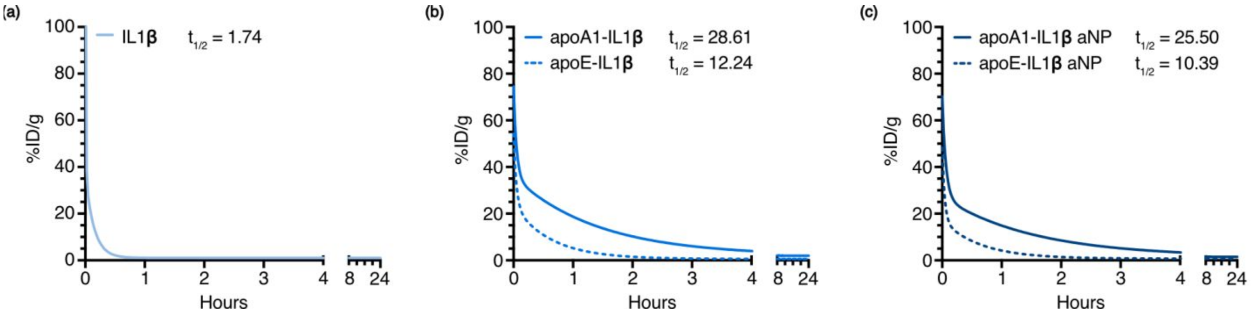

Supplementary Figure 9. Blood half-life measurements for (a) IL1β, (b) IL1β fusion proteins and (c) IL1β-aNPs. Calculated blood half-life is noted in graph.

SUPPLEMENTARY TABLES

Supplementary Table 1: Intensities of bands on SDS-PAGE, determined with ImageJ.

	Unpurified			Purified		
	**IL1β**	**IL2**	**IL4**	**IL1β**	**IL2**	**IL4**
**Conjugate**	5731,463	4007,325	5048,329	11172,6	9020,25	8581,56
**ApoA1**	3466,312	3277,068	3797,262	5894,94	4663,99	3840,98
**Cytokine**	4158,359	5082,145	5542,574	3241,92	3491,15	2770,68
**% conjugate**	42,9	32,4	35,1	55,0	52,5	56,5
**% apoA1**	25,9	26,5	26,4	29,0	27,2	25,3
**% cytokine**	31,1	41,1	38,5	16,0	20,3	18,2

Supplementary Table 2: EC50 values of cytokines and cytokine-ApoA1 conjugation products, derived from HEK-Blue assays displayed in main **Figure 2**.

**Protein**	**EC50 value (nM)**
IL1β	1.85
apoA1-IL1β	20.95
IL2	0.22
apoA1 IL2	0.31
IL4	0.10
apoA1 IL4	0.49

Supplementary Table 3: DNA sequences

**sequence ID**	**DNA sequence (5' to 3')**
apoA1-S230C	ATGCATCACCATCACCATCACGGCCTGGTGCCGCGCGGCAGCATCGATGAT CCGCCGCAGAGTCCATGGGATCGCGTGAAGGACCTGGCCACTGTGTACGTG GATGTGCTCAAAGACAGCGGCAGAGACTATGTGTCTCAGTTTGAAGGATCC

	GCCTTGGGCAAACAATTGAACCTTAAGCTGCTGGACAACTGGGACAGCGTG<br>ACGTCCACCTTCAGCAAGCTGCGCGAACAGCTCGGCCCTGTGACCCAGGAA<br>TTCTGGGATAACCTGGAAAAGGAGACAGAGGGCCTGCGCCAGGAGATGAG<br>CAAGGATCTGGAGGAGGTGAAGGCCAAGGTGCAGCCGTACCTGGACGACT<br>TCCAGAAGAAGTGGCAGGAGGAGATGGAGCTCTACCGCCAGAAGGTGGAG<br>CCGCTGCGCGCAGAGCTGCAGGAGGGCGCGCGCCAGAAGCTGCACGAGCT<br>GCAAGAGAAGCTGAGCCCACTGGGCGAGGAGATGCGCGACCGCGCGCGCG<br>CCCATGTCGACGCGCTGCGCACGCATCTGGCGCCGTACAGCGACGAGCTGC<br>GCCAGCGCTTAGCGGCGCGCCTTGAGGCTCTCAAGGAGAACGGCGGGGCCC<br>GCCTGGCCGAGTACCACGCCAAGGCCACCGAGCATCTGAGCACGCTCAGCG<br>AGAAGGCCAAGCCGGCGCTCGAGGACCTGCGCCAAGGCCTGCTGCCGGTG<br>CTGGAGAGCTTCAAGGTCAGCTTCCTGTGCGCTCTGGAAGAGTACACTAAG<br>AAGCTTAACACCCAGTGA
SUMO-IL1β	ATGGGCGGTAGCCATCACCACCACCACCACGGTAGCATGAGCGACAGCGA<br>AGTGAACCAAGAAGCGAAGCCGGAAGTTAAGCCGGAAGTGAAGCCGGAAA<br>CCCACATCAACCTGAAAGTGAGCGATGGTAGCAGCGAAATCTTCTTTAAGA<br>TTAAGAAAACCACCCCGCTGCGTCGTCTGATGGAGGCGTTCGCGAAGCGTC<br>AGGGTAAAGAAATGGACAGCCTGCGTTTTCTGTACGATGGCATCCGTATTC<br>AGGCGGACCAAACCCCGGAGGACCTGGATATGGAAGACAACGATATCATT<br>GAGGCGCACCGTGAACAGATCGGTGGCGGTACCGCACCAGTGCGCTCCTTG<br>AACTGTACTCTCCGCGACTCGCAGCAGAAGTCCTTGGTGATGAGCGGGCCA<br>TACGAGTTGAAGGCGTTACATTTACAGGGGCAGGACATGGAGCAACAGGTG<br>GTATTCTCTATGTCGTTTGTGCAGGGCGAAGAAAGCAATGATAAGATCCCT<br>GTGGCTTTGGGTTTAAAAGAGAAAAATCTCTATCTCTCATGTGTACTCAAGG<br>ATGATAAACCTACTTTACAGTTAGAAAGCGTAGATCCTAAGAATTACCCAA<br>AGAAAAAAATGGAAAAGCGGTTTGTGTTTAATAAAATTGAGATTAATAATA<br>AATTAGAGTTTGAAAGTGCCCAATTCCCTAACTGGTATATTTCCACCAGTCA<br>GGCTGAGAATATGCCGGTGTTCTTAGGCGGCACCAAGGGTGGTCAGGACAT<br>TACAGACTTTACCATGCAATTCGTATCATCGGGCTCTGGTAGCGGCTCTGGT<br>TCTGGTTCTGGTGCTAGCTGGAGCCATCCGCAGTTTGAGAAATAA
SUMO-IL2	ATGGGCGGTAGCCATCACCACCACCACCACGGTAGCATGAGCGACAGCGA<br>AGTGAACCAAGAAGCGAAGCCGGAAGTTAAGCCGGAAGTGAAGCCGGAAA<br>CCCACATCAACCTGAAAGTGAGCGATGGTAGCAGCGAAATCTTCTTTAAGA<br>TTAAGAAAACCACCCCGCTGCGTCGTCTGATGGAGGCGTTCGCGAAGCGTC<br>AGGGTAAAGAAATGGACAGCCTGCGTTTTCTGTACGATGGCATCCGTATTC<br>AGGCGGACCAAACCCCGGAGGACCTGGATATGGAAGACAACGATATCATT<br>GAGGCGCACCGTGAACAGATCGGTGGCGGTACCGCGCCGACCAGCAGCAG<br>CACCAAGAAGACCCAGCTGCAACTGGAACACCTGCTGCTGGACCTGCAAAT<br>GATTCTGAACGGTATCAACAACTACAAGAACCCGAAACTGACCCGTATGCT<br>GACCTTCAAGTTTTATATGCCGAAGAAAGCGACCGAGCTGAAGCACCTGCA<br>ATGCCTGGAGGAAGAGCTGAAACCGCTGGAAGAGGTGCTGAACCTGGCGC<br>AAAGCAAGAACTTCCACCTGCGTCCGCGTGACCTGATCAGCAACATTAACG<br>TGATCGTTCTGGAACTGAAAGGCAGCGAGACCACCTTTATGTGCGAATATG<br>CGGATGAGACCGCGACCATTGTTGAGTTCCTGAACCGTTGGATCACCTTTTG<br>CCAGAGCATCATTAGCACCCTGACCGGCTCTGGTAGCGGCTCTGGTTCTGGT<br>TCTGGTGCTAGCTGGAGCCATCCGCAGTTTGAGAAATAA

SUMO-IL4	ATGGGCGGTAGCCATCACCACCACCACCACGGTAGCATGAGCGACAGCGA<br>AGTGAACCAAGAAGCGAAGCCGGAAGTTAAGCCGGAAGTGAAGCCGGAAA<br>CCCACATCAACCTGAAAGTGAGCGATGGTAGCAGCGAAATCTTCTTTAAGA<br>TTAAGAAAACCACCCCGCTGCGTCGTCTGATGGAGGCGTTCGCGAAGCGTC<br>AGGGTAAAGAAATGGACAGCCTGCGTTTTCTGTACGATGGCATCCGTATTC<br>AGGCGGACCAAACCCCGGAGGACCTGGATATGGAAGACAACGATATCATT<br>GAGGCGCACCGTGAACAGATCGGTGGCGGTACCGCTAGCCATAAATGTGAT<br>ATTACCCTGCAGGAAATTATTAAAACCCTGAATAGCCTGACCGAACAGAAA<br>ACCCTGTGTACCGAACTGACCGTTACCGATATTTTTGCAGCAAGCAAAAAT<br>ACCACCGAAAAAGAAACCTTTTGTCGTGCAGCAACCGTTCTGCGTCAGTTTT<br>ATAGCCATCATGAAAAAGATACCCGTTGTCTGGGTGCAACCGCACAGCAGT<br>TTCATCGTCATAAACAGCTGATTCGTTTTCTGAAACGTCTGGATCGTAATCT<br>GTGGGGTCTGGCAGGTCTGAATAGCTGTCCGGTTAAAGAAGCAAATCAGAG<br>CACCCTGGAAAATTTTCTGGAACGTCTGAAAACCATTATGCGTGAAAAATA<br>TAGCAAATGTAGCAGCGCTAGCGGTGGTTCTGGTGGTTCTGGTGGTTCTTGG<br>AGCCATCCGCAGTTTGAGAAATAA
ApoA1	ATGCATCACCATCACCATCACGGCCTGGTGCCGCGCGGCAGCATCGATGATCCGCCG<br>CAGAGTCCATGGGATCGCGTGAAGGACCTGGCCACTGTGTACGTGGATGTGCTCAAA<br>GACAGCGGCAGAGACTATGTGTCTCAGTTTGAAGGATCCGCCTTGGGCAAACAATTG<br>AACCTTAAGCTGCTGGACAACTGGGACAGCGTGACGTCCACCTTCAGCAAGCTGCGC<br>GAACAGCTCGGCCCTGTGACCCAGGAATTCTGGGATAACCTGGAAAAGGAGACAGA<br>GGGCCTGCGCCAGGAGATGAGCAAGGATCTGGAGGAGGTGAAGGCCAAGGTGCAGC<br>CGTACCTGGACGACTTCCAGAAGAAGTGGCAGGAGGAGATGGAGCTCTACCGCCAG<br>AAGGTGGAGCCGCTGCGCGCAGAGCTGCAGGAGGGCGCGCGCCAGAAGCTGCACGA<br>GCTGCAAGAGAAGCTGAGCCCACTGGGCGAGGAGATGCGCGACCGCGCGCGCGCCC<br>ATGTCGACGCGCTGCGCACGCATCTGGCGCCGTACAGCGACGAGCTGCGCCAGCGCT<br>TAGCGGCGCGCCTTGAGGCTCTCAAGGAGAACGGCGGGGCCCGCCTGGCCGAGTAC<br>CACGCCAAGGCCACCGAGCATCTGAGCACGCTCAGCGAGAAGGCCAAGCCGGCGCT<br>CGAGGACCTGCGCCAAGGCCTGCTGCCGGTGCTGGAGAGCTTCAAGGTCAGCTTCCT<br>GAGCGCTCTGGAAGAGTACACTAAGAAGCTTAACACCCAGTGA
ApoE	ATGAAAGTCGAGCAAGCCGTAGAAACCGAGCCCGAGCCTGAGTTGCGTCAGCAAAC<br>CGAATGGCAGAGCGGTCAACGCTGGGAACTGGCATTGGGCCGCTTCTGGGATTATCT<br>GCGCTGGGTGCAGACCTTATCAGAGCAAGTACAAGAGGAGTTATTGTCGTCGCAAGT<br>GACGCAGGAGCTTCGTGCCTTAATGGACGAGACGATGAAAGAACTGAAGGCATACA<br>AATCAGAGCTTGAGGAACAATTAACCCCCGTGGCCGAGGAGACACGCGCGCGTTTA<br>TCAAAGGAGTTGCAGGCTGCTCAGGCTCGTTTGGGAGCTGACATGGAAGATGTTCGC<br>GGTCGTCTTGTTCAGTACCGTGGCGAAGTCCAGGCTATGCTGGGCCAGTCAACGGAG<br>GAACTTCGCGTCCGCCTTGCCTCGCATTTACGCAAGTTACGTAAACGTCTTTTGCGCG<br>ACGCTGATGACCTTCAAAAGCGTTTGGCAGTATACCAGGCTGGTGCGCGTGAAGGCG<br>CTGAACGTGGTTTGTCTGCAATCCGTGAGCGTTTGGGTCCACTGGTCGAGCAAGGCC<br>GCGTACGCGCGGCTACTGTTGGGTCACTTGCGGGGCAACCTTTGCAAGAGCGTGCCC<br>AAGCGTGGGGGGAACGTCTTCGTGCCCGCATGGAAGAAATGGGAAGCCGCACGCGC<br>GATCGTCTGGACGAAGTGAAAGAACAAGTTGCAGAGGTTCGCGCAAAACTGGAAGA<br>GCAGGCCCAACAAATTCGTTTGCAAGCTGAGGCGTTTCAGGCGCGCTTGAAGTCATG<br>GTTCGAGCCATTGGTAGAGGACATGCAGCGTCAATGGGCAGGGTTAGTGGAGAAAG<br>TGCAGGCGGCAGTTGGTACTTCTGCTGCCCCAGTGCCATCCGACAATCATGCTAGCC<br>ATCACCATCACCATCATTAA

ApoA1 IL1β	ATGGGTATCCTTCCCAGCCCTGGGATGCCTGCGCTGCTCTCCCTCGTGAGCCTTCTCT<br>CCGTGCTGCTGATGGGTTGCGTAGCTGAAACCGGTTCTGGAAGTGGCCTTGAGGTGC<br>TGTTTCAGGGACCAGGAGGATCCGATGATCCACCTCAAAGTCCATGGGATCGCGTGA<br>AGGACCTGGCCACTGTGTACGTGGATGTGCTCAAAGACAGCGGCAGAGACTATGTGT<br>CTCAGTTTGAAGGCTCCGCCTTGGGCAAACAATTGAACCTTAAGCTGCTGGACAACT<br>GGGACAGCGTGACGTCCACCTTCAGCAAGCTGCGCGAACAGCTCGGCCCTGTGACCC<br>AGGAATTCTGGGATAACCTGGAAAAGGAGACAGAGGGCCTGCGCCAGGAGATGAGC<br>AAGGATCTGGAGGAGGTGAAGGCCAAGGTGCAGCCGTACCTGGACGACTTCCAGAA<br>GAAGTGGCAGGAGGAAATGGAGTTATATCGCCAAAAGGTCGAGCCGCTGCGCGCAG<br>AGCTGCAGGAGGGCGCGCGCCAGAAGCTGCACGAGCTGCAAGAGAAGCTGAGCCCA<br>TTAGGTGAAGAAATGAGAGACCGCGCGCGCGCACATGTCGACGCACTCCGCACACA<br>TCTGGCGCCGTACTCTGACGAGCTGCGCCAGCGCTTAGCGGCGCGCCTTGAGGCTCT<br>CAAGGAGAACGGTGGAGCTCGCCTTGCAGAATATCACGCCAAGGCCACCGAGCATC<br>TGAGCACGCTCAGCGAGAAGGCCAAGCCGGCGCTGGAGGACCTGCGCCAAGGCCTG<br>CTGCCGGTGCTGGAGAGCTTCAAGGTCAGCTTCCTGAGCGCTCTGGAAGAGTACACT<br>AAGAAGCTTAACACCCAGGGTTCTGGTAGCGGCTCTGGTTCTGGTTCTGGTGCACCA<br>GTGCGCTCCTTGAACTGTACTCTCCGCGACTCGCAGCAGAAGTCCTTGGTGATGAGC<br>GGGCCATACGAGTTGAAGGCGTTACATTTACAGGGGCAGGACATGGAGCAACAGGT<br>GGTATTCTCTATGTCGTTTGTGCAGGGCGAAGAAAGCAATGATAAGATCCCTGTGGC<br>TTTGGGTTTAAAAGAGAAAAATCTCTATCTCTCATGTGTACTCAAGGATGATAAACC<br>TACTTTACAGTTAGAAAGCGTAGATCCTAAGAATTACCCAAAGAAAAAAATGGAAA<br>AGCGGTTTGTGTTTAATAAAATTGAGATTAATAATAAATTAGAGTTTGAAAGTGCCC<br>AATTCCCTAACTGGTATATTTCCACCAGTCAGGCTGAGAATATGCCGGTGTTCTTAGG<br>CGGCACCAAGGGTGGTCAGGACATTACAGACTTTACCATGCAATTCGTATCATCGGG<br>TACCGGCAGTGCATGGAGCCATCCACAGTTCGAAAAAGGTGGAGGTTCTGGCGGTG<br>GATCAGGTGGAAGTGCATGGTCTCACCCTCAGTTTGAGAAATAA
ApoE-IL1β	ATGGGTATCCTTCCCAGCCCTGGGATGCCTGCGCTGCTCTCCCTCGTGAGCCTTCTCT<br>CCGTGCTGCTGATGGGTTGCGTAGCTGAAACCGGTTCTGGAAGTGGCCTTGAGGTGC<br>TGTTTCAGGGACCAGGAGGATCCAAAGTCGAGCAAGCCGTAGAAACCGAGCCCGAG<br>CCTGAGTTGCGTCAGCAAACCGAATGGCAGAGCGGTCAACGCTGGGAACTGGCATT<br>GGGCCGCTTCTGGGATTATCTGCGCTGGGTGCAGACCTTATCAGAGCAAGTACAAGA<br>GGAGTTATTGTCGTCGCAAGTGACGCAGGAGCTTCGTGCCTTAATGGACGAGACGAT<br>GAAAGAACTGAAGGCATACAAATCAGAGCTTGAGGAACAATTAACCCCCGTGGCCG<br>AGGAGACACGCGCGCGTTTATCAAAGGAGTTGCAGGCTGCTCAGGCTCGTTTGGGAG<br>CTGACATGGAAGATGTTCGCGGTCGTCTTGTTCAGTACCGTGGCGAAGTCCAGGCTA<br>TGCTGGGCCAGTCAACGGAGGAACTTCGCGTCCGCCTTGCCTCGCATTTACGCAAGT<br>TACGTAAACGTCTTTTGCGCGACGCTGATGACCTTCAAAAGCGTTTGGCAGTATACC<br>AGGCTGGTGCGCGTGAAGGCGCTGAACGTGGTTTGTCTGCAATCCGTGAGCGTTTGG<br>GTCCACTGGTCGAGCAAGGCCGCGTACGCGCGGCTACTGTTGGGTCACTTGCGGGGC<br>AACCTTTGCAAGAGCGTGCCCAAGCGTGGGGGGAACGTCTTCGTGCCCGCATGGAA<br>GAAATGGGAAGCCGCACGCGCGATCGTCTGGACGAAGTGAAAGAACAAGTTGCAGA<br>GGTTCGCGCAAAACTGGAAGAGCAGGCCCAACAAATTCGTTTGCAAGCTGAGGCGT<br>TTCAGGCGCGCTTGAAGTCATGGTTCGAGCCATTGGTAGAGGACATGCAGCGTCAAT<br>GGGCAGGGTTAGTGGAGAAAGTGCAGGCGGCAGTTGGTACTTCTGCTGCCCCAGTGC<br>CATCCGACAATCATGGTTCTGGTAGCGGCTCTGGTTCTGGTTCTGGTGCACCAGTGCG<br>CTCCTTGAACTGTACTCTCCGCGACTCGCAGCAGAAGTCCTTGGTGATGAGCGGGCC<br>ATACGAGTTGAAGGCGTTACATTTACAGGGGCAGGACATGGAGCAACAGGTGGTAT<br>TCTCTATGTCGTTTGTGCAGGGCGAAGAAAGCAATGATAAGATCCCTGTGGCTTTGG

	GTTTAAAAGAGAAAAATCTCTATCTCTCATGTGTACTCAAGGATGATAAACCTACTT<br>TACAGTTAGAAAGCGTAGATCCTAAGAATTACCCAAAGAAAAAAATGGAAAAGCGG<br>TTTGTGTTTAATAAAATTGAGATTAATAATAAATTAGAGTTTGAAAGTGCCCAATTCC<br>CTAACTGGTATATTTCCACCAGTCAGGCTGAGAATATGCCGGTGTTCTTAGGCGGCA<br>CCAAGGGTGGTCAGGACATTACAGACTTTACCATGCAATTCGTATCATCGGGTACCG<br>GCAGTGCATGGAGCCATCCACAGTTCGAAAAAGGTGGAGGTTCTGGCGGTGGATCA<br>GGTGGAAGTGCATGGTCTCACCCTCAGTTTGAGAAATAA
ApoA1 IL2	ATGGGTATCCTTCCCAGCCCTGGGATGCCTGCGCTGCTCTCCCTCGTGAGCCTTCTCT<br>CCGTGCTGCTGATGGGTTGCGTAGCTGAAACCGGTTCTGGAAGTGGCCTTGAGGTGC<br>TGTTTCAGGGACCAGGAGGATCCGATGATCCACCTCAAAGTCCATGGGATCGCGTGA<br>AGGACCTGGCCACTGTGTACGTGGATGTGCTCAAAGACAGCGGCAGAGACTATGTGT<br>CTCAGTTTGAAGGCTCCGCCTTGGGCAAACAATTGAACCTTAAGCTGCTGGACAACT<br>GGGACAGCGTGACGTCCACCTTCAGCAAGCTGCGCGAACAGCTCGGCCCTGTGACCC<br>AGGAATTCTGGGATAACCTGGAAAAGGAGACAGAGGGCCTGCGCCAGGAGATGAGC<br>AAGGATCTGGAGGAGGTGAAGGCCAAGGTGCAGCCGTACCTGGACGACTTCCAGAA<br>GAAGTGGCAGGAGGAAATGGAGTTATATCGCCAAAAGGTCGAGCCGCTGCGCGCAG<br>AGCTGCAGGAGGGCGCGCGCCAGAAGCTGCACGAGCTGCAAGAGAAGCTGAGCCCA<br>TTAGGTGAAGAAATGAGAGACCGCGCGCGCGCACATGTCGACGCACTCCGCACACA<br>TCTGGCGCCGTACTCTGACGAGCTGCGCCAGCGCTTAGCGGCGCGCCTTGAGGCTCT<br>CAAGGAGAACGGTGGAGCTCGCCTTGCAGAATATCACGCCAAGGCCACCGAGCATC<br>TGAGCACGCTCAGCGAGAAGGCCAAGCCGGCGCTGGAGGACCTGCGCCAAGGCCTG<br>CTGCCGGTGCTGGAGAGCTTCAAGGTCAGCTTCCTGAGCGCTCTGGAAGAGTACACT<br>AAGAAGCTTAACACCCAGGGTTCTGGTAGCGGCTCTGGTTCTGGTTCTGGTGCGCCG<br>ACCAGCAGCAGCACCAAGAAGACCCAGCTGCAACTGGAACACCTGCTGCTGGACCT<br>GCAAATGATTCTGAACGGTATCAACAACTACAAGAACCCGAAACTGACCCGTATGCT<br>GACCTTCAAGTTTTATATGCCGAAGAAAGCGACCGAGCTGAAGCACCTGCAATGCCT<br>GGAGGAAGAGCTGAAACCGCTGGAAGAGGTGCTGAACCTGGCGCAAAGCAAGAACT<br>TCCACCTGCGTCCGCGGGACCTGATCAGCAACATTAACGTGATCGTTCTGGAACTGA<br>AAGGCAGCGAGACCACCTTTATGTGCGAATATGCGGATGAGACCGCGACCATTGTTG<br>AGTTCCTGAACCGTTGGATCACCTTTTGCCAGAGCATCATTAGCACCCTGACTGGTAC<br>CGGCAGTGCATGGAGCCATCCACAGTTCGAAAAAGGTGGAGGTTCTGGCGGTGGAT<br>CAGGTGGAAGTGCATGGTCTCACCCTCAGTTTGAGAAATAA

ApoE-IL2	ATGGGTATCCTTCCCAGCCCTGGGATGCCTGCGCTGCTCTCCCTCGTGAGCCTTCTCT<br>CCGTGCTGCTGATGGGTTGCGTAGCTGAAACCGGTTCTGGAAGTGGCCTTGAGGTGC<br>TGTTTCAGGGACCAGGAGGATCCAAAGTCGAGCAAGCCGTAGAAACCGAGCCCGAG<br>CCTGAGTTGCGTCAGCAAACCGAATGGCAGAGCGGTCAACGCTGGGAACTGGCATT<br>GGGCCGCTTCTGGGATTATCTGCGCTGGGTGCAGACCTTATCAGAGCAAGTACAAGA<br>GGAGTTATTGTCGTCGCAAGTGACGCAGGAGCTTCGTGCCTTAATGGACGAGACGAT<br>GAAAGAACTGAAGGCATACAAATCAGAGCTTGAGGAACAATTAACCCCCGTGGCCG<br>AGGAGACACGCGCGCGTTTATCAAAGGAGTTGCAGGCTGCTCAGGCTCGTTTGGGAG<br>CTGACATGGAAGATGTTCGCGGTCGTCTTGTTCAGTACCGTGGCGAAGTCCAGGCTA<br>TGCTGGGCCAGTCAACGGAGGAACTTCGCGTCCGCCTTGCCTCGCATTTACGCAAGT<br>TACGTAAACGTCTTTTGCGCGACGCTGATGACCTTCAAAAGCGTTTGGCAGTATACC<br>AGGCTGGTGCGCGTGAAGGCGCTGAACGTGGTTTGTCTGCAATCCGTGAGCGTTTGG<br>GTCCACTGGTCGAGCAAGGCCGCGTACGCGCGGCTACTGTTGGGTCACTTGCGGGGC<br>AACCTTTGCAAGAGCGTGCCCAAGCGTGGGGGGAACGTCTTCGTGCCCGCATGGAA<br>GAAATGGGAAGCCGCACGCGCGATCGTCTGGACGAAGTGAAAGAACAAGTTGCAGA<br>GGTTCGCGCAAAACTGGAAGAGCAGGCCCAACAAATTCGTTTGCAAGCTGAGGCGT<br>TTCAGGCGCGCTTGAAGTCATGGTTCGAGCCATTGGTAGAGGACATGCAGCGTCAAT<br>GGGCAGGGTTAGTGGAGAAAGTGCAGGCGGCAGTTGGTACTTCTGCTGCCCCAGTGC<br>CATCCGACAATCATGGTTCTGGTAGCGGCTCTGGTTCTGGTTCTGGTGCGCCGACCA<br>GCAGCAGCACCAAGAAGACCCAGCTGCAACTGGAACACCTGCTGCTGGACCTGCAA<br>ATGATTCTGAACGGTATCAACAACTACAAGAACCCGAAACTGACCCGTATGCTGACC<br>TTCAAGTTTTATATGCCGAAGAAAGCGACCGAGCTGAAGCACCTGCAATGCCTGGAG<br>GAAGAGCTGAAACCGCTGGAAGAGGTGCTGAACCTGGCGCAAAGCAAGAACTTCCA<br>CCTGCGTCCGCGTGACCTGATCAGCAACATTAACGTGATCGTTCTGGAACTGAAAGG<br>CAGCGAGACCACCTTTATGTGCGAATATGCGGATGAGACCGCGACCATTGTTGAGTT<br>CCTGAACCGTTGGATCACCTTTTGCCAGAGCATCATTAGCACCCTGACTGGTACCGG<br>CAGTGCATGGAGCCATCCACAGTTCGAAAAAGGTGGAGGTTCTGGCGGTGGATCAG<br>GTGGAAGTGCATGGTCTCACCCTCAGTTTGAGAAATAA
ApoA1 IL4	ATGGGTATCCTTCCCAGCCCTGGGATGCCTGCGCTGCTCTCCCTCGTGAGCCTTCTCT<br>CCGTGCTGCTGATGGGTTGCGTAGCTGAAACCGGTTCTGGAAGTGGCCTTGAGGTGC<br>TGTTTCAGGGACCAGGAGGATCCGATGATCCACCTCAAAGTCCATGGGATCGCGTGA<br>AGGACCTGGCCACTGTGTACGTGGATGTGCTCAAAGACAGCGGCAGAGACTATGTGT<br>CTCAGTTTGAAGGCTCCGCCTTGGGCAAACAATTGAACCTTAAGCTGCTGGACAACT<br>GGGACAGCGTGACGTCCACCTTCAGCAAGCTGCGCGAACAGCTCGGCCCTGTGACCC<br>AGGAATTCTGGGATAACCTGGAAAAGGAGACAGAGGGCCTGCGCCAGGAGATGAGC<br>AAGGATCTGGAGGAGGTGAAGGCCAAGGTGCAGCCGTACCTGGACGACTTCCAGAA<br>GAAGTGGCAGGAGGAAATGGAGTTATATCGCCAAAAGGTCGAGCCGCTGCGCGCAG<br>AGCTGCAGGAGGGCGCGCGCCAGAAGCTGCACGAGCTGCAAGAGAAGCTGAGCCCA<br>TTAGGTGAAGAAATGAGAGACCGCGCGCGCGCACATGTCGACGCACTCCGCACACA<br>TCTGGCGCCGTACTCTGACGAGCTGCGCCAGCGCTTAGCGGCGCGCCTTGAGGCTCT<br>CAAGGAGAACGGTGGAGCTCGCCTTGCAGAATATCACGCCAAGGCCACCGAGCATC<br>TGAGCACGCTCAGCGAGAAGGCCAAGCCGGCGCTGGAGGACCTGCGCCAAGGCCTG<br>CTGCCGGTGCTGGAGAGCTTCAAGGTCAGCTTCCTGAGCGCTCTGGAAGAGTACACT<br>AAGAAGCTTAACACCCAGGGTTCTGGTAGCGGCTCTGGTTCTGGTTCTGGTCATAAA<br>TGTGATATTACCCTGCAGGAAATTATTAAAACCCTGAATAGCCTGACCGAACAGAAA<br>ACCCTGTGTACCGAACTGACCGTTACCGATATTTTTGCAGCAAGCAAAAATACCACC<br>GAAAAAGAAACCTTTTGTCGTGCAGCAACCGTTCTGCGTCAGTTTTATAGCCATCAT<br>GAAAAAGATACCCGTTGTCTGGGTGCAACCGCACAGCAGTTTCATCGTCATAAACAG

	CTGATTCGTTTTCTGAAACGTCTGGATCGTAATCTGTGGGGTCTGGCAGGTCTGAATA<br>GCTGTCCGGTTAAAGAAGCAAATCAGAGCACCCTGGAAAATTTTCTGGAACGTCTGA<br>AAACCATTATGCGTGAAAAATATAGCAAATGTAGCAGCGGTACCGGCAGTGCATGG<br>AGCCATCCACAGTTCGAAAAAGGTGGAGGTTCTGGCGGTGGATCAGGTGGAAGTGC<br>ATGGTCTCACCCTCAGTTTGAGAAATAA
ApoE-IL4	ATGGGTATCCTTCCCAGCCCTGGGATGCCTGCGCTGCTCTCCCTCGTGAGCCTTCTCT<br>CCGTGCTGCTGATGGGTTGCGTAGCTGAAACCGGTTCTGGAAGTGGCCTTGAGGTGC<br>TGTTTCAGGGACCAGGAGGATCCAAAGTCGAGCAAGCCGTAGAAACCGAGCCCGAG<br>CCTGAGTTGCGTCAGCAAACCGAATGGCAGAGCGGTCAACGCTGGGAACTGGCATT<br>GGGCCGCTTCTGGGATTATCTGCGCTGGGTGCAGACCTTATCAGAGCAAGTACAAGA<br>GGAGTTATTGTCGTCGCAAGTGACGCAGGAGCTTCGTGCCTTAATGGACGAGACGAT<br>GAAAGAACTGAAGGCATACAAATCAGAGCTTGAGGAACAATTAACCCCCGTGGCCG<br>AGGAGACACGCGCGCGTTTATCAAAGGAGTTGCAGGCTGCTCAGGCTCGTTTGGGAG<br>CTGACATGGAAGATGTTCGCGGTCGTCTTGTTCAGTACCGTGGCGAAGTCCAGGCTA<br>TGCTGGGCCAGTCAACGGAGGAACTTCGCGTCCGCCTTGCCTCGCATTTACGCAAGT<br>TACGTAAACGTCTTTTGCGCGACGCTGATGACCTTCAAAAGCGTTTGGCAGTATACC<br>AGGCTGGTGCGCGTGAAGGCGCTGAACGTGGTTTGTCTGCAATCCGTGAGCGTTTGG<br>GTCCACTGGTCGAGCAAGGCCGCGTACGCGCGGCTACTGTTGGGTCACTTGCGGGGC<br>AACCTTTGCAAGAGCGTGCCCAAGCGTGGGGGGAACGTCTTCGTGCCCGCATGGAA<br>GAAATGGGAAGCCGCACGCGCGATCGTCTGGACGAAGTGAAAGAACAAGTTGCAGA<br>GGTTCGCGCAAAACTGGAAGAGCAGGCCCAACAAATTCGTTTGCAAGCTGAGGCGT<br>TTCAGGCGCGCTTGAAGTCATGGTTCGAGCCATTGGTAGAGGACATGCAGCGTCAAT<br>GGGCAGGGTTAGTGGAGAAAGTGCAGGCGGCAGTTGGTACTTCTGCTGCCCCAGTGC<br>CATCCGACAATCATGGTTCTGGTAGCGGCTCTGGTTCTGGTTCTGGTCATAAATGTGA<br>TATTACCCTGCAGGAAATTATTAAAACCCTGAATAGCCTGACCGAACAGAAAACCCT<br>GTGTACCGAACTGACCGTTACCGATATTTTTGCAGCAAGCAAAAATACCACCGAAAA<br>AGAAACCTTTTGTCGTGCAGCAACCGTTCTGCGTCAGTTTTATAGCCATCATGAAAA<br>AGATACCCGTTGTCTGGGTGCAACCGCACAGCAGTTTCATCGTCATAAACAGCTGAT<br>TCGTTTTCTGAAACGTCTGGATCGTAATCTGTGGGGTCTGGCAGGTCTGAATAGCTGT<br>CCGGTTAAAGAAGCAAATCAGAGCACCCTGGAAAATTTTCTGGAACGTCTGAAAAC<br>CATTATGCGTGAAAAATATAGCAAATGTAGCAGCGGTACCGGCAGTGCATGGAGCC<br>ATCCACAGTTCGAAAAAGGTGGAGGTTCTGGCGGTGGATCAGGTGGAAGTGCATGG<br>TCTCACCCTCAGTTTGAGAAATAA

Supplementary Table 4: Amino acid sequences of proteins produced in E. Coli.

**sequence ID**	**Amino acid sequence**

apoA1-S230C	MHHHHHHGLVPRGSIDDPPQSPWDRVKDLATVYVDVLKDSGRDYVSQFEGSA<br>LGKQLNLKLLDNWDSVTSTFSKLREQLGPVTQEFWDNLEKETEGLRQEMSKD<br>LEEVKAKVQPYLDDFQKKWQEEMELYRQKVEPLRAELQEGARQKLHELQEKL<br>SPLGEEMRDRARAHVDALRTHLAPYSDELRQRLAARLEALKENGGARLAEYH<br>AKATEHLSTLSEKAKPALEDLRQGLLPVLESFKVSFLCALEEYTKKLNTQ*
SUMO-IL1β	MGGSHHHHHHGSMSDSEVNQEAKPEVKPEVKPETHINLKVSDGSSEIFFKIKKT<br>TPLRRLMEAFAKRQGKEMDSLRFLYDGIRIQADQTPEDLDMEDNDIIEAHREQI<br>GGGTAPVRSLNCTLRDSQQKSLVMSGPYELKALHLQGQDMEQQVVFSMSFVQ<br>GEESNDKIPVALGLKEKNLYLSCVLKDDKPTLQLESVDPKNYPKKKMEKRFVF<br>NKIEINNKLEFESAQFPNWYISTSQAENMPVFLGGTKGGQDITDFTMQFVSSGS<br>GSGSGSGSGASWSHPQFEK*
SUMO-IL2	MGGSHHHHHHGSMSDSEVNQEAKPEVKPEVKPETHINLKVSDGSSEIFFKIKKT<br>TPLRRLMEAFAKRQGKEMDSLRFLYDGIRIQADQTPEDLDMEDNDIIEAHREQI<br>GGGTAPTSSSTKKTQLQLEHLLLDLQMILNGINNYKNPKLTRMLTFKFYMPKK<br>ATELKHLQCLEEELKPLEEVLNLAQSKNFHLRPRDLISNINVIVLELKGSETTFM<br>CEYADETATIVEFLNRWITFCQSIISTLTGSGSGSGSGSGASWSHPQFEK*
SUMO-IL4	MGGSHHHHHHGSMSDSEVNQEAKPEVKPEVKPETHINLKVSDGSSEIFFKIKKT<br>TPLRRLMEAFAKRQGKEMDSLRFLYDGIRIQADQTPEDLDMEDNDIIEAHREQI<br>GGGTASHKCDITLQEIIKTLNSLTEQKTLCTELTVTDIFAASKNTTEKETFCRAA<br>TVLRQFYSHHEKDTRCLGATAQQFHRHKQLIRFLKRLDRNLWGLAGLNSCPV<br>KEANQSTLENFLERLKTIMREKYSKCSSASGGSGGSGGSWSHPQFEK*

ApoA1 SUMO-tag His6-tag GGS linker Cytokine Strep-tag

Supplementary table 5. Amino acid sequences and expected molecular weights of proteins produced in HEK293S cells.

Protein	Predicted MW (Da)	Amino acid sequence
ApoA1	29799	MHHHHHHGLVPRGSIDDPPQSPWDRVKDLATVYVDVLKDSGRDY<br>VSQFEGSALGKQLNLKLLDNWDSVTSTFSKLREQLGPVTQEFWDN<br>LEKETEGLRQEMSKDLEEVKAKVQPYLDDFQKKWQEEMELYRQK<br>VEPLRAELQEGARQKLHELQEKLSPLGEEMRDRARAHVDALRTHL<br>APYSDELRQRLAARLEALKENGGARLAEYHAKATEHLSTLSEKAK<br>PALEDLRQGLLPVLESFKVSFLSALEEYTKKLNTQ*
ApoE	35402	MKVEQAVETEPEPELRQQTEWQSGQRWELALGRFWDYLRWVQTL<br>SEQVQEELLSSQVTQELRALMDETMKELKAYKSELEEQLTPVAEET<br>RARLSKELQAAQARLGADMEDVRGRLVQYRGEVQAMLGQSTEEL<br>RVRLASHLRKLRKRLLRDADDLQKRLAVYQAGAREGAERGLSAIR<br>ERLGPLVEQGRVRAATVGSLAGQPLQERAQAWGERLRARMEEMG<br>SRTRDRLDEVKEQVAEVRAKLEEQAQQIRLQAEAFQARLKSWFEP<br>LVEDMQRQWAGLVEKVQAAVGTSAAPVPSDNHASHHHHHH*

ApoA1 IL1β	53870	MGILPSPGMPALLSLVSLLSVLLMGCVAETGSGSGLEVLFQGPGGSDDPPQSPWDRVKDLATVYVDVLKDSGRDYVSQFEGSALGKQLNLKLLDNWDSVTSTFSKLREQLGPVTQEFWDNLEKETEGLRQEMSKDLEEVKAKVQPYLDDFQKKWQEEMELYRQKVEPLRAELQEGARQKLHELQEKLSPLGEEMRDRARAHVDALRTHLAPYSDELRQRLAARLEALKENGGARLAEYHAKATEHLSTLSEKAKPALEDLRQGLLPVLESFKVSFLSALEEYTKKLNTQGSGSGSGSGSGAPVRSLNCTLRDSQQKSLVMSGPYELKALHLQGQDMEQQVVFSMSFVQGEESNDKIPVALGLKEKNLYLSCVLKDDKPTLQLESVDPKNYPKKKMEKRFVFNKIEINNKLEFESAQFPNWYISTSQAENMPVFLGGTKGGQDITDFTMQFVSSGTGSAWSHPQFEKGGGSGGGSGGSAWSHPQFEK*
ApoE-IL1β	60095	MGILPSPGMPALLSLVSLLSVLLMGCVAETGSGSGLEVLFQGPGGSKVEQAVETEPEPELRQQTEWQSGQRWELALGRFWDYLRWVQTLSEQVQEELLSSQVTQELRALMDETMKELKAYKSELEEQLTPVAEETRARLSKELQAAQARLGADMEDVRGRLVQYRGEVQAMLGQSTEELRVRLASHLRKLRKRLLRDADDLQKRLAVYQAGAREGAERGLSAIRERLGPLVEQGRVRAATVGSLAGQPLQERAQAWGERLRARMEEMGSRTRDRLDEVKEQVAEVRAKLEEQAQQIRLQAEAFQARLKSWFEPLVEDMQRQWAGLVEKVQAAVGTSAAPVPSDNHGSGSGSGSGSGAPVRSLNCTLRDSQQKSLVMSGPYELKALHLQGQDMEQQVVFSMSFVQGEESNDKIPVALGLKEKNLYLSCVLKDDKPTLQLESVDPKNYPKKKMEKRFVFNKIEINNKLEFESAQFPNWYISTSQAENMPVFLGGTKGGQDITDFTMQFVSSGTGSAWSHPQFEKGGGSGGGSGGSAWSHPQFEK*
ApoA1 IL2	51911	MGILPSPGMPALLSLVSLLSVLLMGCVAETGSGSGLEVLFQGPGGSDDPPQSPWDRVKDLATVYVDVLKDSGRDYVSQFEGSALGKQLNLKLLDNWDSVTSTFSKLREQLGPVTQEFWDNLEKETEGLRQEMSKDLEEVKAKVQPYLDDFQKKWQEEMELYRQKVEPLRAELQEGARQKLHELQEKLSPLGEEMRDRARAHVDALRTHLAPYSDELRQRLAARLEALKENGGARLAEYHAKATEHLSTLSEKAKPALEDLRQGLLPVLESFKVSFLSALEEYTKKLNTQGSGSGSGSGSGAPTSSSTKKTQLQLEHLLLDLQMILNGINNYKNPKLTRMLTFKFYMPKKATELKHLQCLEEELKPLEEVLNLAQSKNFHLRPRDLISNINVIVLELKGSETTFMCEYADETATIVEFLNRWITFCQSIISTLTGTGSAWSHPQFEKGGGSGGGSGGSAWSHPQFEK*
ApoE-IL2	58136	MGILPSPGMPALLSLVSLLSVLLMGCVAETGSGSGLEVLFQGPGGSKVEQAVETEPEPELRQQTEWQSGQRWELALGRFWDYLRWVQTLSEQVQEELLSSQVTQELRALMDETMKELKAYKSELEEQLTPVAEETRARLSKELQAAQARLGADMEDVRGRLVQYRGEVQAMLGQSTEELRVRLASHLRKLRKRLLRDADDLQKRLAVYQAGAREGAERGLSAIRERLGPLVEQGRVRAATVGSLAGQPLQERAQAWGERLRARMEEMGSRTRDRLDEVKEQVAEVRAKLEEQAQQIRLQAEAFQARLKSWFEPLVEDMQRQWAGLVEKVQAAVGTSAAPVPSDNHGSGSGSGSGSGAPTSSSTKKTQLQLEHLLLDLQMILNGINNYKNPKLTRMLTFKFYMPKKATELKHLQCLEEELKPLEEVLNLAQSKNFHLRPRDLISNINVIVLELKGSETTFMCEYADETATIVEFLNRWITFCQSIISTLTGTGSAWSHPQFEKGGGSGGGSGGSAWSHPQFEK*
ApoA1 IL4	51456	MGILPSPGMPALLSLVSLLSVLLMGCVAETGSGSGLEVLFQGPGGSDDPPQSPWDRVKDLATVYVDVLKDSGRDYVSQFEGSALGKQLNLKLLDNWDSVTSTFSKLREQLGPVTQEFWDNLEKETEGLRQEMSKDLEEVKAKVQPYLDDFQKKWQEEMELYRQKVEPLRAELQEGARQKLHELQEKLSPLGEEMRDRARAHVDALRTHLAPYSDELRQRLAARLEALKENGGARLAEYHAKATEHLSTLSEKAKPALEDLRQGLLPVLESFKVSFLSALEEYTKKLNTQGSGSGSGSGSGHKCDITLQEIIKTLNSLTEQKTLCTELTVTDIFAASKNTTEKETFCRAATVLRQFYSHHEKDTRCLGATAQQFHRHKQLIRFLKRLDRNLWGLAGLNSCPVKEANQSTLENFLERLKTIMREKYSKCSSGTGSAWSHPQFEKGGGSGGGSGGSAWSHPQFEK*

ApoE-IL4	57681	MGILPSPGMPALLSLVSLLSVLLMGCVAETGSGSGLEVLFQGPGGS<br>KVEQAVETEPEPELRQQTEWQSGQRWELALGRFWDYLRWVQTLS<br>EQVQEELLSSQVTQELRALMDETMKELKAYKSELEEQLTPVAEET<br>RARLSKELQAAQARLGADMEDVRGRLVQYRGEVQAMLGQSTEEL<br>RVRLASHLRKLRKRLLRDADDLQKRLAVYQAGAREGAERGLSAIR<br>ERLGPLVEQGRVRAATVGSLAGQPLQERAQAWGERLRARMEEMG<br>SRTRDRLDEVKEQVAEVRAKLEEQAQQIRLQAEAFQARLKSWFEP<br>LVEDMQRQWAGLVEKVQAAVGTSAAPVPSDNHGSGSGSGSGSGH<br>KCDITLQEIIKTLNSLTEQKTLCTELTVTDIFAASKNTTEKETFCRAA<br>TVLRQFYSHHEKDTRCLGATAQQFHRHKQLIRFLKRLDRNLWGLA<br>GLNSCPVKEANQSTLENFLERLKTIMREKYSKCSSGTGSAWSHPQF<br>EKGGGSGGGSGGSAWSHPQFEK*

Secretion tag   Purification tag   Cleavage tag   Apolipoprotein   Cytokine   Linker

Supplementary table 5. EC50 values of cytokines, fusion proteins and aNPs, determined by HEKBlue assays displayed in main **Figure 3 and 4**.

**Protein or aNP**	**EC50 value (nM)**
IL1β	0.03
apoA1 IL1β	5.49
apoE-IL1β	1.32
apoA1-IL1β aNP	33.74
apoE-IL1β aNP	7.96
IL2	0.09
apoA1 IL2	0.01
apoE-IL2	0.03
apoA1-IL2 aNP	0.27
apoE-IL2 aNP	0.17
IL4	0.02
apoA1 IL4	2.63
apoE-IL4	4.90
apoA1-IL4 aNP	5.68
apoE-IL4 aNP	21.78
